# Oryzines A & B, Maleidride Congeners from *Aspergillus oryzae* and Their Putative Biosynthesis

**DOI:** 10.3390/jof4030096

**Published:** 2018-08-13

**Authors:** Zahida Wasil, Eric Kuhnert, Thomas J. Simpson, Russell J. Cox

**Affiliations:** 1 University of Bristol, School of Chemistry, Cantock’s Close, Bristol BS8 1TS, UK; zahida.wasil@gmail.com; 2 Leibniz Universität Hannover, Biomolekulares Wirkstoffzentrum und Institut für Organische Chemie, Schneiderberg 38, 30167 Hannover, Germany; eric.kuhnert@oci.uni-hannover.de; 3 University of Bristol, School of Chemistry, Cantock’s Close, Bristol BS8 1TS, UK; Tom.Simpson@bristol.ac.uk; 4 Leibniz Universität Hannover, Biomolekulares Wirkstoffzentrum und Institut für Organische Chemie, Schneiderberg 38, 30167 Hannover, Germany

**Keywords:** natural products, citrate synthase, biosynthetic gene cluster, structure elucidation, fatty acid synthase

## Abstract

*Aspergillus oryzae* is traditionally used in East Asia for the production of food and brewing. In addition, it has been developed into a suitable host for the heterologous expression of natural product biosynthetic genes and gene clusters, enabling the functional analysis of the encoded enzymes. *A. oryzae* shares a 99.5% genome homology with *Aspergillus flavus*, but their secondary metabolomes differ significantly and various compounds unique to *A. oryzae* have been reported. While using *A. oryzae* as a host for heterologous expression experiments we discovered two new metabolites in extracts of *A. oryzae* M-2-3 with an unusual maleidride backbone, which were named oryzine A and B. Their structures were elucidated by high resolution mass spectrometry (HRMS) and nuclear magnetic resonance (NMR) analysis. Their structural relationships with known maleidrides implied involvement of a citrate synthase (CS) and a polyketide (PKS) or fatty acid synthase (FAS) in their biosynthesis. Analysis of the *A. oryzae* genome revealed a single putative biosynthetic gene cluster (BGC) consistent with the hypothetical biosynthesis of the oryzines. These findings increase knowledge of the chemical potential of *A. oryzae* and are the first attempt to link a novel product of this fungus with genomic data.

## 1. Introduction

*Aspergillus oryzae* is a filamentous fungus widely used in the food production industry of many East Asian countries to ferment soy beans as basis for soy sauces, miso and fermented bean paste, but also for brewing of various beverages [1]. In addition, enzymes used in food processing, such as α-amylases, aminopeptidases, lactases, lipases or proteases, are isolated in large scale from *A. oryzae* [2]. Due to its economic importance the fungus has been extensively studied and genetic tools became readily available to manipulate the organism. In combination with its advantageously limited set of native compounds *A. oryzae* has been successfully developed as a heterologous expression system for fungal secondary metabolites [2,3]. This system has been important in the discovery and functional analysis of various biosynthetic gene clusters (BGC) including xenovulenes, pleuromutilins, maleidrides and aphidicolin [4,5,6,7].

Whole genome sequencing of the RIB40 strain of *A. oryzae* and other *Aspergillus* species revealed a 99.5% similarity between the genomes of *Aspergillus flavus* and *A. oryzae*. Therefore, it has been suggested that the latter is a domesticated variety of *A. flavus*, mainly differing in the lack of aflatoxin production [8]. An in-depth chemical comparison between the two species showed striking differences in the metabolites produced. Thus, *A. oryzae* produces kojic acid, the diterpene indoles aflavinines, the aflatrem precursor 13-desoxypaxilline, the sesquiterpenes parasiticolides and various alkaloids, e.g., ditryptoleucine and oryzamides [9]. Other compounds produced by *A. oryzae* have been reported recently [10,11,12]. However, identifications of the respective producing strains were solely based on morphological characteristics and ribosomal genes, which are insufficient to clearly differentiate them from *A. flavus* [13]. Even though the genome sequence of *A. oryzae* has been available since 2005 [14], only the BGCs of aflatoxin, cyclopiazonic acid, aflatrem, kojic acid, 3, 5-dihydroxybenzoic acid and penicillin have been identified [9,15,16,17]. This is a small number compared with the 32 annotated polyketide synthase (PKS) and 27 annotated non-ribosomal peptide synthetase (NRPS) BGCs of *A. oryzae*, and leaves much room for new findings [9].

In this study, we report the identification and structure elucidation of two new secondary metabolites of the maleidride family produced by *A. oryzae* M-2-3 [18]. In addition, a candidate BGC encoded by the genome of *A. oryzae* is bioinformatically characterized and a putative biosynthetic pathway is presented.

## 2. Materials and Methods 

### 2.1. General

Optical rotations were determined with an ADP 220 polarimeter at 589 nm and IR spectra were recorded with a Perkin–Elmer FTIR instrument. NMR spectra were recorded on a Varian VNMRS-500 spectrometer (^1^H 500 MHz, ^13^C 125 MHz). HR-ESI-MS mass spectra were determined with a Bruker Daltonics Apex IV FT-ICR. HPLC analysis was performed on a Waters 2795HT HPLC system. Detection was achieved by UV between 200 and 400 nm using a Waters 998 diode array detector, and by simultaneous electrospray (ES) mass spectrometry using a Waters ZQ spectrometer detecting between 150 and 600 *m/z* units. Chromatography (flow rate 1 mL/min) was performed using a Phenomenex Kinetex column (2.6 μ, C_18_, 100 Å, 4.6 × 100 mm) equipped with a Phenomenex Security Guard precolumn (Luna C_5_ 300 Å). The mobile phase consisted of solvent A (HPLC grade H_2_O containing 0.05% formic acid) and solvent B (HPLC grade CH_3_CN containing 0.045% formic acid) subjected to the following gradient: 0 min, 10% B; 10 min, 90% B; 12 min, 90% B; 13 min, 10% B; 15 min, 10% B.

### 2.2. Fermentation and Extraction

Three liters of *A. oryzae* M-2-3 pTAex3-tenS(8309)·egfp [19] (30 flasks × 100 mL) were grown in production medium (20 g/L starch, 10 g/L peptone, 2 g/L NaNO_3_, 2 g/L KCl, 1 g/L K_2_HPO_4_, 0.5 g MgSO_4_.7H_2_O, 0.01 g FeSO_4_.7H_2_O) for 6 days at 28 °C and shaken at 200 rpm. Cells and media were homogenized using a hand-held electric blender and then acidified to pH 4.0 using 37% aqueous HCl. An equal volume of ethyl acetate was added and the mixture was stirred for 10 min. and then vacuum filtered through Whatman no. 1 filter paper. The filtrate was transferred into a separating funnel and shaken vigorously. The organic layer was washed once with concentrated brine solution and then with deionized water. The organic phase was dried (MgSO_4_), filtered and evaporated to dryness. The crude extract was dissolved in 10% aqueous methanol and defatted by extraction with hexane. The methanolic layer was evaporated to dryness to yield crude extract (239 mg) and analyzed by liquid chromatography mass spectrometry (LCMS).

### 2.3. Isolation of Oryzine A **3** and Oryzine B **4**

The crude extract was dissolved in HPLC grade methanol and subjected to mass-directed preparative HPLC. Purification of compounds was achieved using a Waters mass-directed auto-purification system comprising of a Waters 2767 autosampler, Waters 2545 pump system, a Phenomenex LUNA column (5 μ, C_18_, 100 Å, 10 × 250 mm) equipped with a Phenomenex Security Guard precolumn (Luna C_5_ 300 Å) eluted at 16 mL/min. Solvents used were: A, HPLC grade H_2_O + 0.05% formic acid; B, HPLC grade CH_3_CN + 0.045% formic acid. The post-column flow was split (100:1) and the minority flow was made up with solvent A to 1 mL/min for simultaneous analysis by diode array detector (Waters 2998; Milford, MA, USA), evaporative light scattering (Waters 2424; Milford, MA, USA) and ESI mass spectrometry in positive and negative modes (Waters Quatro Micro; Milford, MA, USA). The gradient started with 40% to 80% B over 15 min, continued for 0.5 min to 95% and ended with 1 min isocratic conditions. Fractions of 24 consecutive runs were combined to yield 9.5 mg of **3** and 9.0 mg of **4**.

### 2.4. Spectroscopic and Spectrometric Data

The corresponding 1D and 2D NMR spectra of **3** and **4** are depicted in the Supplementary Information (Figures S1–S10).

#### 2.4.1. Oryzine A **3**

Light brown viscous oil, [α]^22^_D_ −16.9 (*c* = 0.23, MeOH); IR (neat): ν_max_ 2932, 2872, 2342, 1761, 1631, 1355, 1190, 1084 cm^−1^; ^1^H NMR (CDCl_3_, 500 MHz) *δ* = 0.92 (3H, t, *J* = 7 Hz, H-1), 1.36 (2 H, m, H-2), 1.37 (1 H, m, H-3a), 1.45 (1 H, m, H-3b), 1.65 (1 H, m, H-4a), 1.81 (1 H, m, H-4b), 2.01 (1 H, ddd, *J* = 12, 10.5, 12 Hz, H-6a), 2.55 (1 H, ddd, *J* = 9, 6, 12 Hz, H-6b), 3.65 (1H, t, *J* = 9, 12 Hz, H-7), 4.43 (1 H, m, H-5), 5.95 (1 H, b, H-10a), 6.54 (1 H, b, H-10b), ^13^C NMR (CDCl_3_, 125 MHz) *δ* = 14.4 (C-1), 22.9 (C-2), 27.8 (C-3), 35.5 (C-4), 36.1 (C-6), 45.2 (C-7), 79.6 (C-5), 131.8 (C-10), 136.1 (C-8), 170.1(C-9), 176.4 (C-11). HRESIMS calculated for C_11_H_17_O_4_: 213.1121; observed 213.1130 [M + H]^+^.

#### 2.4.2. Oryzine B **4**

Light color viscous oil, IR (neat): ν_max_ 2962, 2930, 2873, 1736, 1582, 1454, 1045, 878 cm^−1^; ^1^H NMR (CDCl_3_, 500 MHz) *δ* = 0.92 (3 H, t, *J* = 7 Hz, H-1), 1.38 (2 H, m, H-2), 1.46 (2 H, m, H-3), 1.71 (1 H, m, H-4a), 1.79 1 H, m, H-4b), 4.99 (1 H, t, *J* = 6.2 Hz, H-5), 6.79 (1 H, b, H-10a), 7.19 (1 H, b, H-10b), 7.96 (1 H, b, H-6); ^13^C NMR (CDCl_3_, 125 MHz) *δ* = 14.0 (C-1), 22.6 (C-2), 27.3 (C-3), 33.1 (C-4), 80.8 (C-5), 125.0 (C-7), 128.5 (C-8), 133.6 (C-10), 153.5 (C-6), 169.5 (C-9), 171.7 (C-11). HRESIMS calculated for C_11_H_14_O_4_Na: 233.0784; observed 233.0799 [M + Na]^+^.

### 2.5. Oryzine Biosynthetic Gene Cluster Prediction 

The prediction of the BGC responsible for oryzine production is based on the genome information available for *A. oryzae* RIB40 (BioProject: PRJNA28175). The producing strain of the oryzines (M-2-3) is an auxotrophic mutant derived from *A. oryzae* RIB203, which is genetically very close to RIB40 (https://www.nrib.go.jp/data/asp/strain.html). Due to annotation gaps a part of the genome was re-annotated. Gene prediction was accomplished by applying AUGUSTUS version 3.3. [20] on the *A. oryzae* RIB40 chromosome 8 (RefSeq: NC_036442.1) using the precomputed gene model for this species. For comparison purpose, in addition, GeneMark-ES version 4.3.3. [21] was applied by using the parameters for self-training (–ES) and fungal genomes (–fungus). Clustered genes in *A. oryzae* related to the byssochlamic acid BGC from *Byssochlamys fulva* were identified with the MultiGeneBlast architecture search [22] using *bfpks1*, *bfl2* and *bfl3* [6] as templates. Homologies of the putative oryzine BGC with related BGCs from *A. flavus* NRRL 3357 and *B. fulva* were analyzed with the Artemis comparison tool (ACT) [23].

## 3. Results

**Figure 1:**
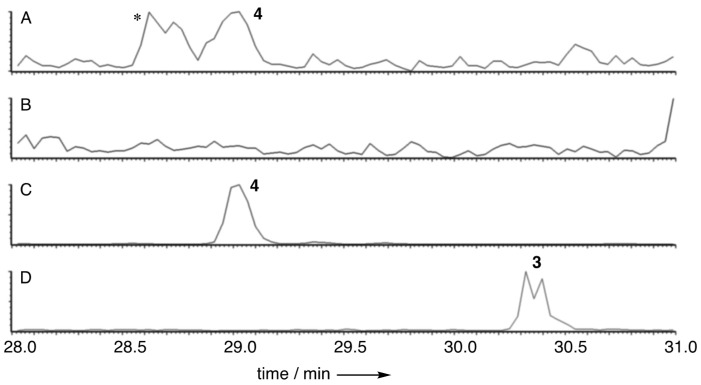
LCMS analysis of extracts of *A. oryzae*. (**A**) WT *A. oryzae* M-2-3, extracted ion chromatogram (EIC^+^) for *m/z* 213.1 (oryzine B **4**, [M + H]^+^); (**B**) WT *A. oryzae* M-2-3, EIC^+^ for *m/*z 211.1 (oryzine A **3**, [M + H]^+^); (**C**) *A. oryzae* M-2-3 pTAex3-tenS(8309)·egfp EIC^+^ for *m/z* 213.1 (oryzine B **4**, [M + H]^+^); (**D**) *A. oryza*e M-2-3 pTAex3-tenS(8309)·egfp EIC+ for *m/z* 211.1 (oryzine A **3**, [M + H]^+^). * unrelated compound.

As part of a larger project involving the use of *A. oryzae* M-2-3 [18] as a host for the heterologous expression of engineered fungal polyketide synthase genes, we were interested in the biosynthesis of pretenellin A 1. This compound is produced by the tenellin synthetase (TENS) which is a hybrid polyketide synthase non-ribosomal peptide synthetase (PKS-NRPS) which works with a *trans*-acting enoyl reductase, encoded by *tenC* or *dmbC* [24,25]. We engineered a system containing a truncated *tenS* gene, lacking the sequence encoding the NRPS, together with *dmbC* [19]. We predicted that this would produce the tenellin polyketide 2 with nominal mass 210 Da. Comparison of transformed (Figure 1C,D) vs WT *A. oryzae* M-2-3 (Figure 1A,B) revealed the presence of a new compound (RT 30.3 min) in the transformed strain with the expected nominal mass (Figure 1D, [M + H]^+^ 211.1). This was accompanied by a compound (RT 29.0 min, [M + H]^+^ 213.1) which is present in both WT and transformed strains, although at significantly lower titer in the WT strain (<1%). Both compounds were purified from extracts of the transformed *A. oryzae* strain by mass-directed HPLC. Full structure elucidation (*vide infra*) revealed these two compounds to be unrelated to pretenellin A 1, showing that the original experiment had failed.

### 3.1. Structure Elucidation

**Figure 2:**
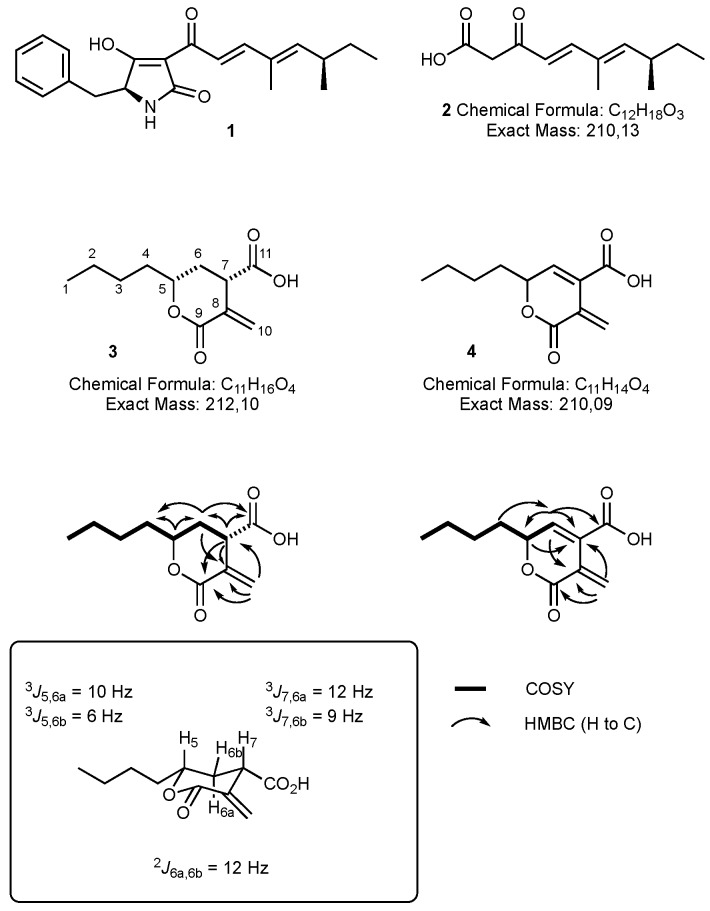
Chemical structures of pretenellin A **1** and the predicted product **2** of the tenellin PKS. Actual structures of oryzine A **3** and B **4**.

Oryzine A **3** was obtained as light brown viscous oil. The HRESIMS data of **3** indicated a molecular formula of C_11_H_17_O_4_ (calc. 213.1121, obs. 213.1130, [M + H]^+^). The carbon spectra (125 MHz) revealed the presence of eleven carbons, which included two carbonyl groups at δ_C_ 176.4 (C-11) and 170.1 (C-9), four methylene groups in the alkane region of the spectra and one methyl carbon at δ_C_ 14.4 (C-1, Figure 2). The HSQC (heteronuclear single quantum coherence spectroscopy) spectrum revealed 8 protonated carbons. The ^1^H NMR spectrum showed two distinct doublets in the alkene region at δ_H_ 5.95 and δ_H_ 6.54 which were assigned to two geminal methylene protons attached to C-10. One of these methylene protons shows HMBC (heteronuclear multiple bond correlation) correlation to an alkene carbon at δ_C_ 136.1 which was assigned C-8. Both geminal methylene protons (H-10) show HMBC correlations to C-9 and a methine at δ_C_ 45.2 (C-7). The triplet at δ_H_ 3.65 was assigned to a methine proton (H-7) which showed an HMBC correlation to C-11, C-9 and an alkene carbon at δ_C_ 136.1 (C-8). H-7 shows COSY (correlation spectroscopy) coupling to two geminal methylene protons at δ_H_ 2.01 and 2.55 (H-6). These, in turn, show COSY and HMBC connections to a methine proton at δ_H_ 4.43 (C-5) attached to an oxygen atom. The chemical shift of δ_C_ 79.6 for C-5 supports its link to an oxygen atom. This data suggested the presence of a pyrone with a carboxylic acid at C-7. The methine protons at δ_H_ 4.43 (H-5) and methylene protons at δ_H_ 2.01 and 2.55 (H-6) show COSY connections to two other geminal protons at δ_H_ 1.65 and 1.81 (H-4). Two multiplets at δ_H_ 1.37 and 1.46 were assigned to geminal methylene protons (3-CH_2_) which displays a COSY coupling to one of the methylenes at δ_H_ 1.8 (H-4). The broad signal at δ_H_ 1.35–1.39 was assigned to methylene protons (2-CH_2_) showing correlations to δ_C_ 27.8 (C-3) in the HMBC spectrum and to the methyl at δ_H_ 0.92 (H-1). Both HMBC and COSY correlations show an aliphatic chain (C1-C4) attached to the pyran at C-5 methine.

The relative orientation of the protons at the two stereocentres C-5 and C-7 was established using the coupling constants of H-5 and H-7 with their adjacent methylene protons at δ_H_ 2.01 (H-6a) and δ_H_ 2.55 (H-6b, Figure 2). A J value of 6 Hz between H-5 and H-6b indicated this pair of protons to be gauche, while the J value of 10 Hz between H-5 and H-6a suggesting this pair as anti and thus axial/axial. The methine proton H-7 displayed a J value of 12 Hz with H-6a and 9 Hz with H-6b. These values suggest that H-7 is also axial.

The structure of **4** was assigned using HRMS and NMR analysis and by comparison to **3**. The chemical formula is C_11_H_14_O_4_ (HRMS, observed 233.0799; calculated 233.0784 for [M + Na]^+^). The ^13^C NMR displayed the presence of 11 carbons including two carbonyls at δ_C_ 169.5 (C-9) and 171.7 (C-11), a methine carbon at δ_C_ 80.8 (C-5) attached to an oxygen atom, a methyl group at δ_C_ 14.0 (C-1), three methylene groups and four olefinic carbons at δ_C_ 125.0 (C-7), 128.5 (C-8), 133.6 (C-10) and 153.5 (C-6, Figure 2). The HSQC spectrum showed seven protonated carbons. In the ^1^H NMR, the broad doublet signal downfield at δ_H_ 7.96 was assigned to the methine (H-6) attached to the olefinic carbon at δ_C_ 153.5 (C-6) in the HSQC spectrum. In the HMBC spectrum, H-6 shows linkage to methine C-5 and to the olefinic carbon C-7. This confirmed the presence of an olefin between C-6 and C-7. H-6 also shows HMBC correlations with the olefin carbons C-8 and C-11. The two broad resonances at δ_H_ 6.79 and 7.19 were assigned to geminal methylene protons attached to C-10 showing correlations to the methine protons H-5 and H-6 in the COSY, and HMBC correlations to the olefin carbon C-7 and to the C-9 carbonyl. The triplet signal at δ_H_ 4.99 was assigned to the H-5 methine, displaying HMBC correlations with the methylene groups at δ_C_ 27.3 (C-3) and 33.1 (C-4) and to olefin carbons C-7 and C-6. The two multiplet signals at δ_H_ 1.71 and 1.79 were assigned to geminal methylene protons (H-4) correlated to C-2 and C-3 in the HMBC. The methyl signal at δ_H_ 0.92 (H-1) showed HMBC correlations to the methylene groups at C-2 and C-3. This compound was thus assigned the same carbon skelton as oryzine A **3**, but unsaturated between C-6 and C-7.

### 3.2. Identification of A Putative Oryzine Biosynthetic Gene Cluster

**Figure 3:**
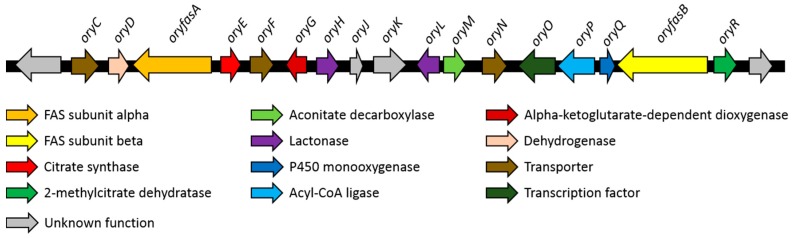
Putative biosynthetic gene cluster of the oryzines.

The structures of the oryzines indicate a biosynthetic relationship to the maleidrides [6,26,27]. In general, the biosynthesis of maleidrides is characterized by condensation of an acyl CoA thiolester of variable chain-length with oxaloacetic acid, catalyzed by a citrate synthase (CS). In addition, a 2-methyl citrate dehydratase (2MCD) is required to catalyze subsequent steps [6]. Therefore, the *A. oryzae* genome was analyzed for a BGC containing genes encoding those enzymes. MultiGeneBlast [22] analysis (Figure S11) indicated the presence of a single BGC that contains genes encoding a CS and a 2MCD. The respective genes had no PKS in their proximity but were instead surrounded by α and β subunits of a fungal FAS. To verify that this gene cluster is the only possible candidate, a manual search of the *A. oryzae* genome for CS genes was also conducted. A total of five homologues of CS were identified on the *A. oryzae* genome by using the CS (Bfl2) of the byssochlamic acid BGC (ANF07286) from *Byssochlamys fulva* as template. The genes in the proximity to these five targets were analyzed in detail. Four of these genes (amino acid sequence identity with Bfl2 between 55.5% and 11.2%) were not close to any recognized types of biosynthetic genes. The fifth gene (37.7% amino acid sequence identity) was the one predicted by MultiGeneBlast. In addition to the 2MCD and FAS subunits various other biosynthetic genes including oxygenases, lactonases and a decarboxylase were found in this region of the genome. Hence, this BGC was considered as the most likely candidate for oryzine biosynthesis (Figure 3).

### 3.3. Characteristics of the Putative Oryzine BGC

The putative oryzine BGC has a size of around 47 kb and contains 18 predicted genes (Table 1, Table S1). The two largest genes (*oryfasA*, *oryfasB*) encode the α- and β-subunits of a fungal fatty acid synthase (FAS). Additional copies of FAS genes can be found within the genome of *A. oryzae. OryfasA* is located at the downstream part of the BGC next to the citrate synthase (*oryE*) and a putative dehydrogenase (*oryD*). In contrast, *oryfasB* is found at the upstream end of the BGC flanked by a 2MCD (*oryR*) and a gene encoding a protein with high similarity to sterigmatocystin P450 monooxygenase (*oryQ*). The short amino acid sequence of OryQ (170 aa) implies a truncation, probably hampering its functionality. Between the two FAS encoding genes additional biosynthetic genes can be identified. OryG is homologous to the α -ketoglutarate-dependent taurine dioxygenase (P37610, 31% identity) and OryH as well as OryL contain a domain that belongs to the lactonase superfamily. *OryM* encodes a protein related to aconitate decarboxylases, whereas *oryP* is most likely encodes an acyl-CoA ligase. In addition, multiple putative transporters (*oryC*, *oryF*, *oryN*) and a putative transcription factor (*oryP*) can be found. The genes *oryJ* and *oryK* cannot be assigned to any known biosynthetic family.

### 3.4. Proposed Biosynthesis of the Oryzines

**Figure 4:**
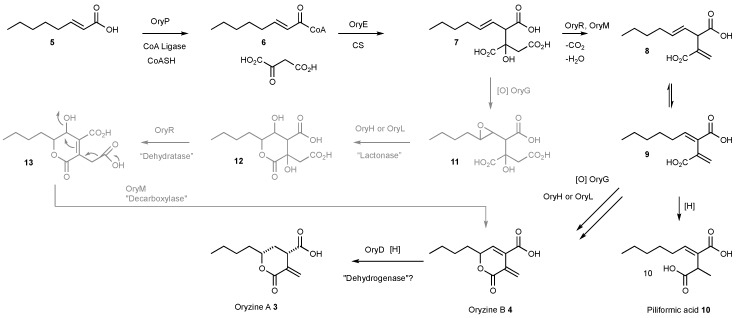
Proposed biosynthetic pathway for oryzine A **3** and B **4**.

With the set of identified biosynthetic genes in hand, a putative biosynthetic pathway can be proposed (Figure 4). The two subunits of the fungal FAS (OryfasA, OryfasB) probably form octenoic acid **5**. This fatty acid is most likely activated by the acyl-CoA ligase OryP to give octenyl CoA **6** before the CS (OryE) acts to catalyze condensation with oxaloacetate to form tricarboxylic acid **7**. At this stage, two pathways appear feasible. In the first, decarboxylation and concomitant dehydration (OryM), followed by tautomerization gives the diene **9**. Reduction of this could give the known metabolite piliformic acid **10** from the fungus *Xylaria mali* which has been shown to be derived from FAS-derived octanoic acid and oxaloacetate by feeding studies [28]. On the pathway to **3** and **4**, however, hydroxylation of the diene **9** (OryG) and lactonisation (OryH or OryL) could give oryzine B **4** directly. Final enoyl reduction (OryD) would then give Oryzine A **3**. An alternative pathway branching from **7** could involve epoxidation to **11** (OryG) followed by ring closure (OryH or OryL). The resulting diol **12** could be a substrate for the MCDH to give unsaturated alcohol **13**. Finally, concerted decarboxylation and dehydration (OryM) could give the observed **4**. We do not yet have enough experimental evidence to distinguish these possibilities, but the first pathway is preferred due to the proximity to the known metabolite piliformic acid **10**.

## 4. Discussion

It is clear that our original genetic engineering experiment in *A. oryzae* M-2-3 did not give the anticipated result. However, the transformed organism produced two compounds, oryzines A **3** and B **4**, in titers significantly higher than untransformed strains of *A. oryzae*. As various other transformants did not show increased production of oryzines it can be speculated that the regulation of gene expression within the oryzine BGC was influenced by the random insertion of the heterologous expressed *tenS* gene into the *A. oryzae* genome. Related compounds such as piliformic acid **10** are known to be derived from fatty acids and oxaloacetate [28]; while the furofuranones 4-*epi*-ethiosiloide **14**, sporothriolide **15** and discosiolide **16**, are also thought to be derived from fatty acids and oxaloacetate [29,30]. These compounds differ from **3** and **10** by having differing carbon-chain lengths, and by a different lactonisation pattern. It is therefore likely that the oryzines, piliformic acid **10** and the furofuranones **14**–**16** are assembled by similar biosynthetic machinery. However, to date, none of these compounds have been linked to any BGC. The presence of a fatty acyl and an oxaloacetyl moiety is also known in the maleidride class of secondary metabolites where a key citrate synthase-type enzyme is responsible for their formation. We thus suspected that the oryzines are a class of hitherto unrecognised maleidrides and the biosynthesis should also require a citrate synthase step.

**Figure 5:**
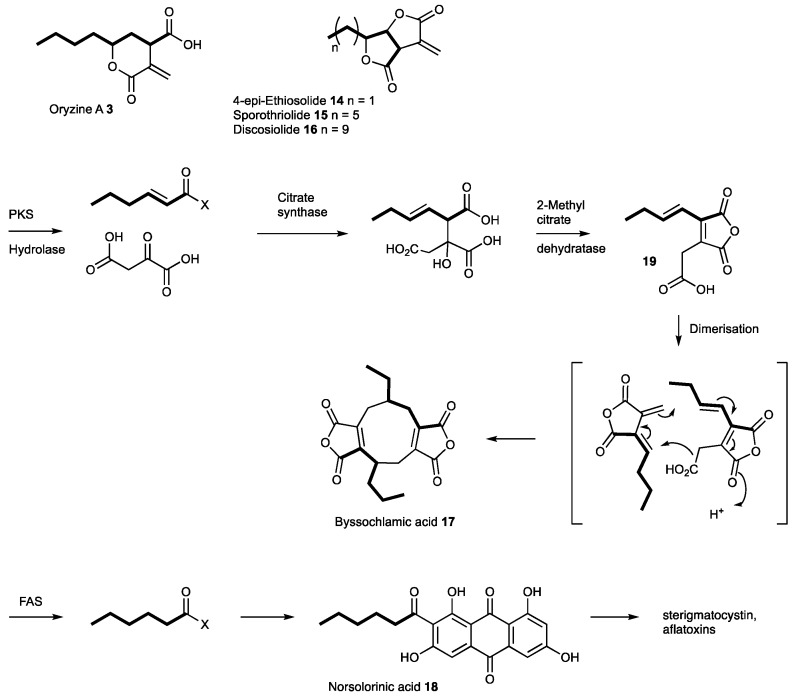
Compounds biosynthetically related to **3**.

Based on the BGC prediction, the first steps of oryzine biosynthesis are likely to be similar to those of the elucidated byssochlamic acid (BA **17**) biosynthesis (Figure 5) [6]. The main difference is the mode of production of the alkenoic acid: in case of BA **17** this is produced by a polyketide synthase; but in the case of **3** and **4** the alkanoic acid is probably produced by a fatty acid synthase. FAS components of fungal BGC are known, but their essential role for the biosynthesis of secondary metabolites has only been partially demonstrated. In case of the aflatoxins, sterigmatocystin and dothistromin the FAS subunits provide the initial hexanoyl starter unit for the PKS, which forms norsolorinic acid **18** [31,32] (Figure 5). A homology search of FAS genes in the genome of *A. oryzae* indicated the presence of four additional copies of both subunits. Only one of these gene pairs had no biosynthetic genes in their proximity, therefore the respective proteins are likely part of primary metabolism. The second FAS copy belongs to the previously identified aflatoxin BGC [31]. The third homologous pair is nested inside an NRPS gene cluster that shows high similarity to the aspercryptin BGC from *A. nidulans* which is known to require octanoic acid during its biosynthesis [33]. The last FAS is part of an azaphilone-type BGC similar to those identified from various *Monascus* and *Talaromyces* (*Penicillium*) species [34]. The high diversity of FAS containing secondary metabolite gene clusters in *A. oryzae* is striking and highlights the importance of such proteins for natural product biosynthesis. 

The BA **17** system requires a hydrolase encoded by *bfl1*, presumably to release the polyketide from the PKS at the end of its synthesis [6]. However in the case of fungal FAS a dedicated acyl transferase, which is an integral component, releases the fatty acid at the end of biosynthesis. Interestingly it has been shown that these AT domains can be engineered to release short fatty acids [35]. Consistent with this idea is the fact that the proposed oryzine cluster does not contain a gene homologous to *bfl1*. Similarly, the BA BGC contains genes which encode proteins responsible for the dimerisation (to form nonadrides such as **17**) which are not present in the proposed oryzine BGC; and oryzine biosynthesis requires oxidative steps and thus the oryzine BGC has encoded oxygenases not present in the BA BGC (Figure S12).

It is likely that maleic acid anhydride intermediates such as **19** (Figure 5) are not formed during oryzine biosynthesis due to the presence of an aconitate decarboxylase, which is not present in the other known maleidride gene clusters. The loss of a carboxyl functionality at C-10 would prevent anhydride formation, which explains the structural differences between the oryzines and the maleic acid anhydrides. In other systems one could imagine oxidations and lactonisations with different regioselectivities leading to the furofuranones **14**–**16**.

Even though only a limited set of biosynthetic steps is necessary to explain the assembly of the oryzines, the BGC seems rather large. However, in order to correctly define the borders of the cluster transcription studies should be conducted. It is also questionable if all predicted genes encode functional proteins. A BGC comparison with *A. flavus* revealed the presence of a homologous BGC with >99.5% similarity (Figure S12), highlighting the potential of *A. flavus* to produce oryzines as well. Whether these compounds have been overlooked in the past by researchers, or are not produced under the tested conditions or whether small mutations in the *A. flavus* BGC abolished production of **3** and **4** or closely related compounds must also be evaluated in the future. 

## 5. Conclusions

The oryzines isolated from *Aspergillus oryzae* constitute a novel class of maleidrides, which are structurally related to furofuranones **14**–**16**. In our previous work we have proven the links between fungal gene clusters by either knockout or heterologous expression in engineered strains of *A. oryzae*. Here, however, the heterologous expression strategy is obviated by the presence of the genes of interest in the expression host itself; while knockout strategies were frustrated by the low titer of **3** and **4** in untransformed *A. oryzae*. However, comparison of the BGCs of *A. oryzae* with the byssochlamic acid gene cluster from *B. fulva* revealed a single candidate cluster containing FAS, citrate synthase, dehydratase, decarboxylase and oxygenase genes, and no other likely BGCs in the *A. oryzae* genome. The presence of a FAS and a CS in a single cluster and their link to a final product is shown for the first time, adding to our knowledge about fungal biosynthetic pathways. These observations also allow the development of a firm hypotheses for the biosynthesis (and hence the composition of biosynthetic gene clusters) of the furofuranones and related compounds such as piliformic acid **10**. Further progress, however, will have to await full genome sequencing of the organisms responsible for the biosynthesis of these compounds. Future work will involve the development of new methods to understand such clusters.

## Figures and Tables

**Table 1:** Predicted genes of the oryzine BGC and their proposed function.

Gene	GenBank Acc. No.	Proposed Function	Closest UniProtKB Hit (Query Coverage/Identity)
*oryC*	XP_023094066	Transporter	Sugar transporter STL1, *Saccharomyces cerevisiae*, P39932 (94%/33%)
*oryD*	-	Dehydrogenase	Histidinol dehydrogenase 1, *Bacillus clausii*, Q5WIU9 (99%/56%)
*oryfasA*	XP_001827206	Fatty acid synthase subunit alpha	Fatty acid synthase subunit alpha, *Aspergillus nidulans*, Q5B7V0 (99%/46%)
*oryE*	XP_001827205	Citrate synthase	Citrate synthase, *Dictyostelium discoideum*, Q86AV6 (97%/38%)
*oryF*	XP_001827204	Transporter	Efflux pump VrtL, *Penicillium aethiopicum*, D7PHY8 (95%/39%)
*oryG*	XP_001827203	Alpha-ketoglut- -arate dependent dioxygenase	Alpha-ketoglutarate-dependent sulfonate dioxygenase, *Saccharomyces cerevisiae*, Q12358 (88%/32%)
*oryH*	XP_023094065	Lactonase	Gluconolactonase, *Zymomonas mobilis*, Q01578 (52%/28%)
*oryJ*	XP_023094064	-	Lysine--tRNA ligase, *Bacillus cereus*, B7JK84 (24%/30%)
*oryK*	XP_001827200	-	Ankyrin repeat and KH domain-containing protein mask, *Drosophila melanogaster*, Q9VCA8 (36%/27%)
*oryL*	XP_023094061	Lactonase	Gluconolactonase, *Zymomonas mobilis*, Q01578 (66%/25%)
*oryM*	XP_001827196	Aconitate decarboxylase	Cis-aconitate decarboxylase, *Aspergillus terre*us, Q0C8L3 (98%/55%)
*oryN*	XP_001827195	Transporter	Citrinin biosynthesis cluster MFS transporter Mrr1, *Monascus ruber*, A0A161CLJ6 (93%/48%)
*oryO*	XP_023094060	Transcription factor	Uncharacterized transcriptional regulatory protein C530.05, *Schizosaccharomyces pombe*, O59741 (24%/40%)
*oryP*	-	Acyl-CoA ligase	Acyl-CoA synthetase family member 3, *Homo sapiens*, Q4G176 (92%/23%)
*oryQ*	-	P450 monooxygenase (truncated)	Probable sterigmatocystin biosynthesis P450 monooxygenase StcF, *Aspergillus nidulans*, Q12609 (82%/49%)
*oryfasB*	XP_001827193	Fatty acid synthase subunit beta	Fatty acid synthase subunit beta, *Yarrowia lipolytica*, P34229 (99%/40%)
*oryR*	XP_001827192	2-Methylcitrate dehydratase	2-methylcitrate dehydratase, *Escherichia coli*, P77243 (96%/49%)

## Supplementary Materials

The following are available online at http://www.mdpi.com/2309-608X/4/3/96/s1: Figures S1–S10, 1D and 2D NMR data for **3** and **4**; Figure S11, MultiGeneBlast (architecture search) of the byssochlamic acid biosynthetic genes *bfpks1*, *bfl2* and *bfl3* against the genomes of *Aspergillus oryzae* RIB40 and *A. flavus* NRRL3357; Figure S12, Artemis comparison tool (ACT) analysis between the putative oryzine biosynthetic gene cluster (BGC) of *A. flavus* (A) and *A. oryzae* (B) and the byssochlamic acid BGC of *Byssochlamys fulva* (C); Table S1, Predicted genes and corresponding nucleotide as well as protein sequences of the re-annotated oryzine BGC from *A. oryzae* RIB40.
