# Supplementary material for: Oryzines A & B, Maleidride Congeners from Aspergillus oryzae and Their Putative Biosynthesis
Source: J Fungi (Basel). 2018 Aug 13;4(3):96. doi: 10.3390/jof4030096 (PMC6162547; doi:10.3390/jof4030096)
Supplement: Supplementary file 1 [file jof-04-00096-s001.pdf]

Oryzines A & B, maleidride congeners from *Aspergillus oryzae* and their putative biosynthesis

Zahida Wasil[1], Eric Kuhnert[2] Thomas J. Simpson[1] and Russell J. Cox [1,2,*]

[1] University of Bristol, School of Chemistry, Cantock's Close, Bristol, BS8 1TS, UK;
[2] BMWZ and OCI, Leibniz Universität Hannover, 30167, Hannover, Germany;
* Correspondence: russell.cox@oci.uni-hannover.de

Supplementary Information

1. Spectroscopic Data for 3 and 4.

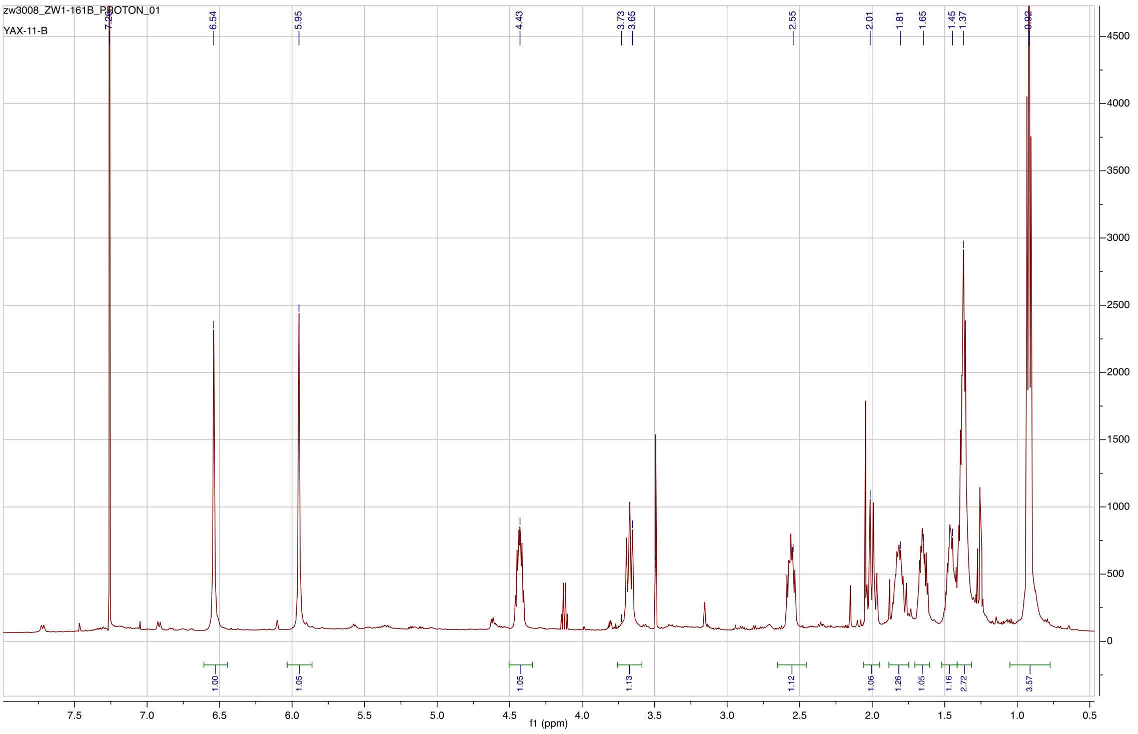

Figure S1. ^{1}H NMR of **3**, 500 MHz.

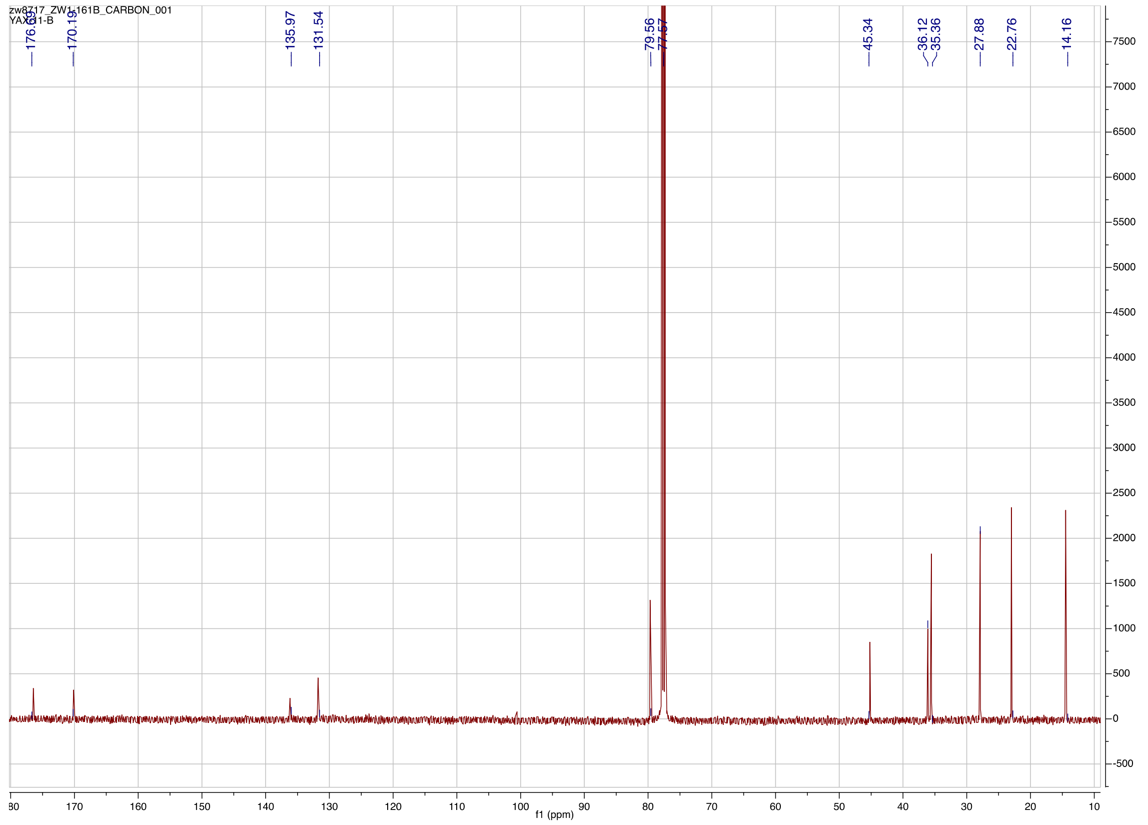

Figure S2. ^{13}C NMR of **4** 125 MHz.

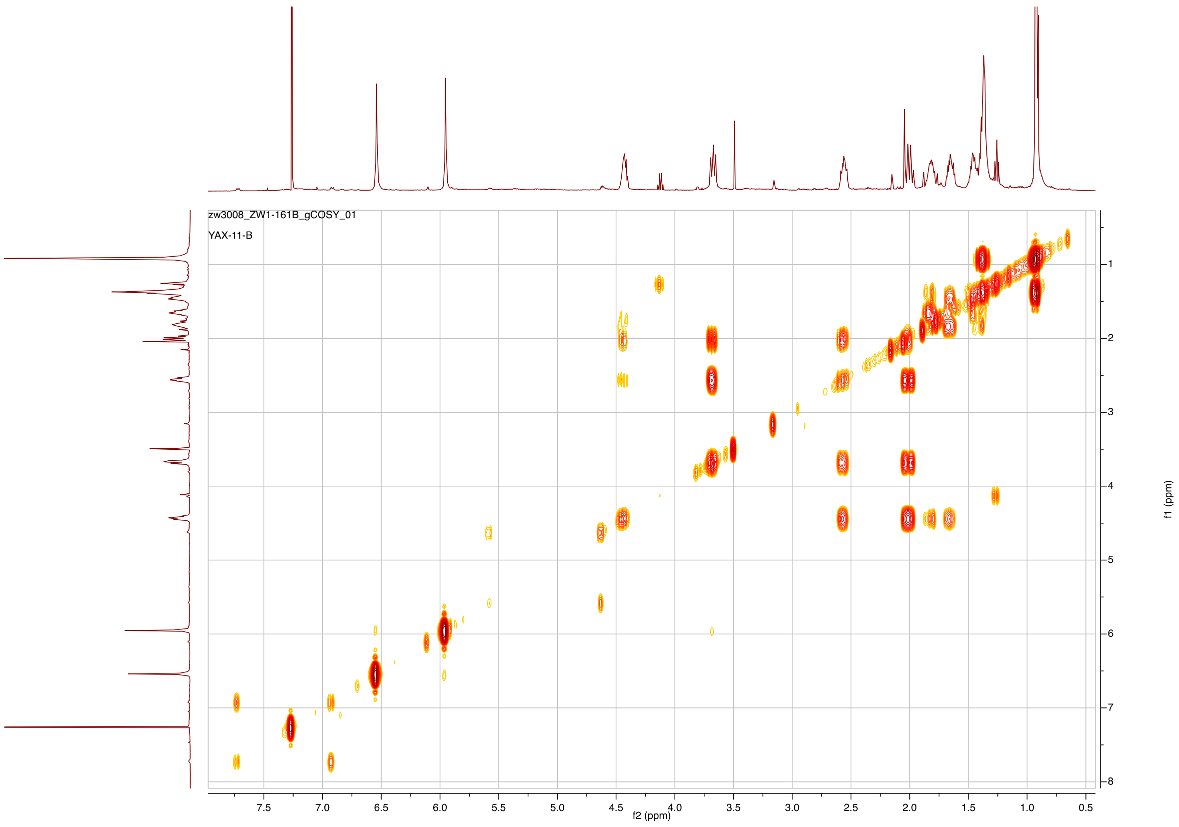

Figure S3. gCOSY of **4.**

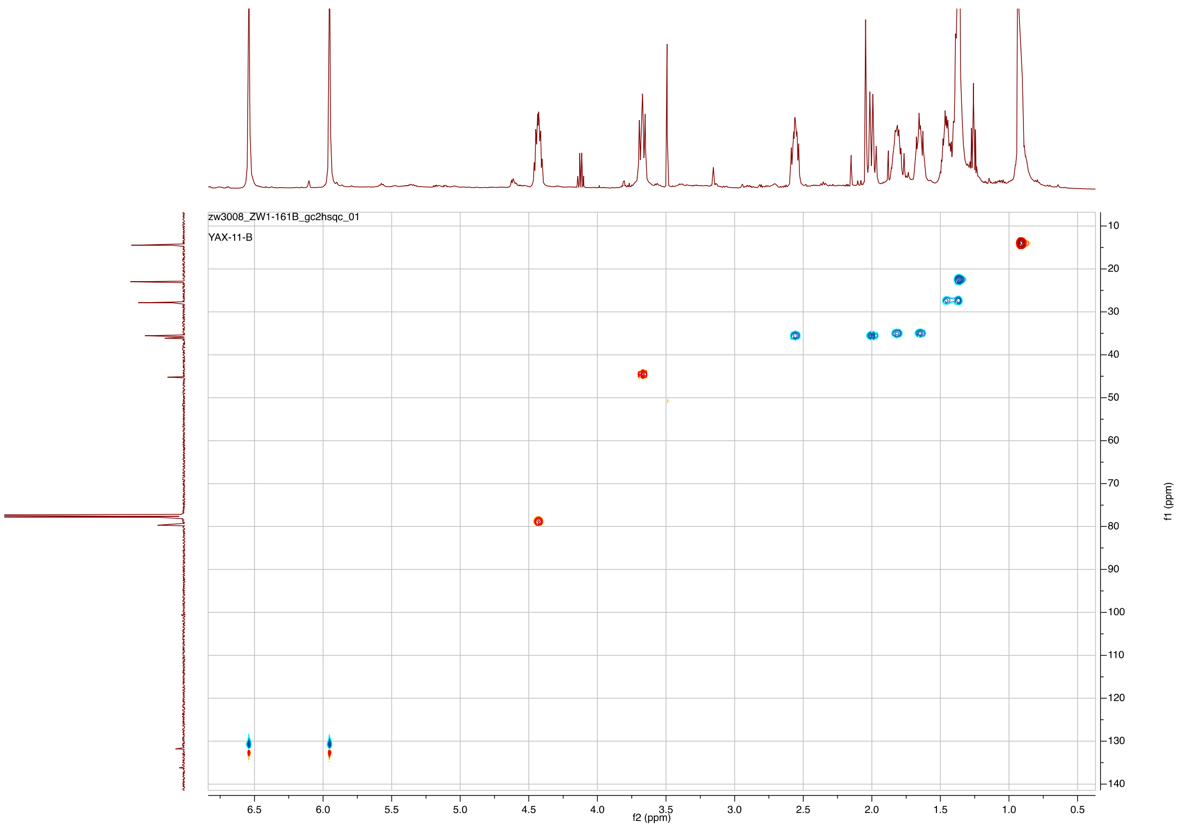

Figure S4. HSQC of **4.**

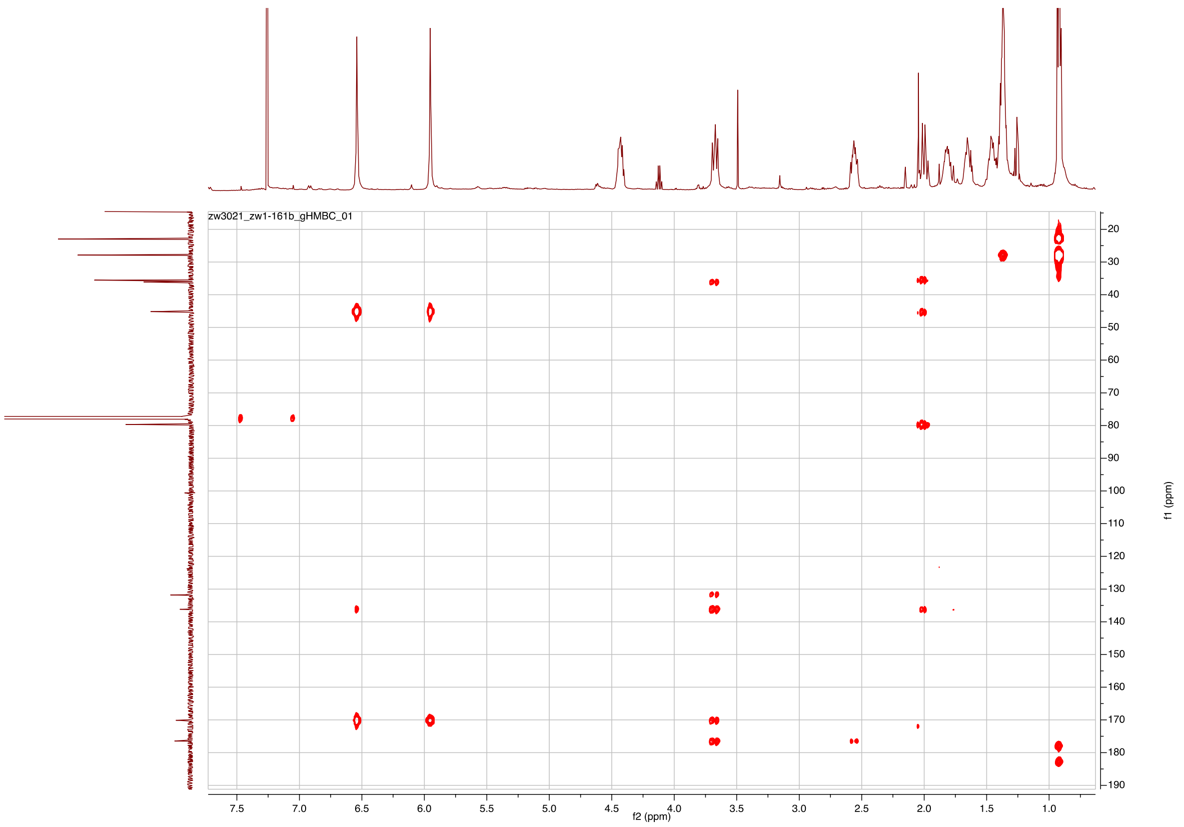

Figure S5. HMBC of **4.**

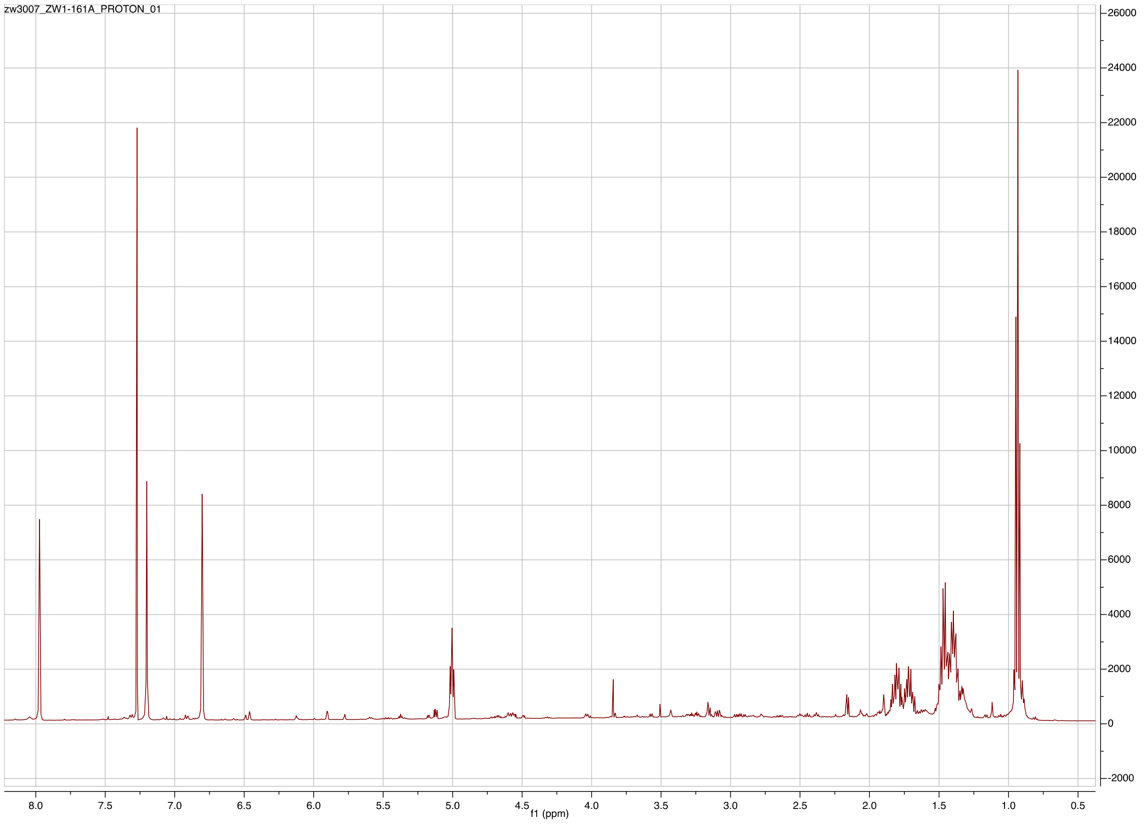

Figure S6. ^{1}H NMR of **4**, 500 MHz.

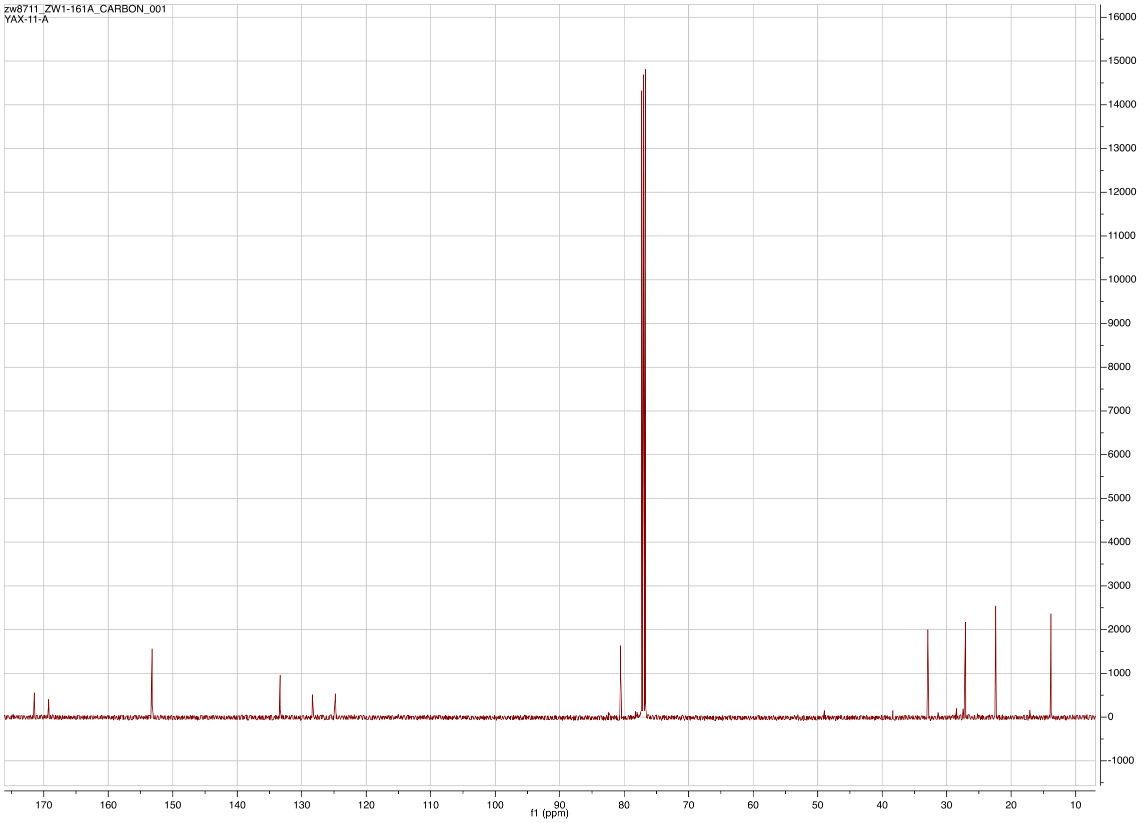

Figure S7. ^{13}C NMR of **4**, 125 MHz.

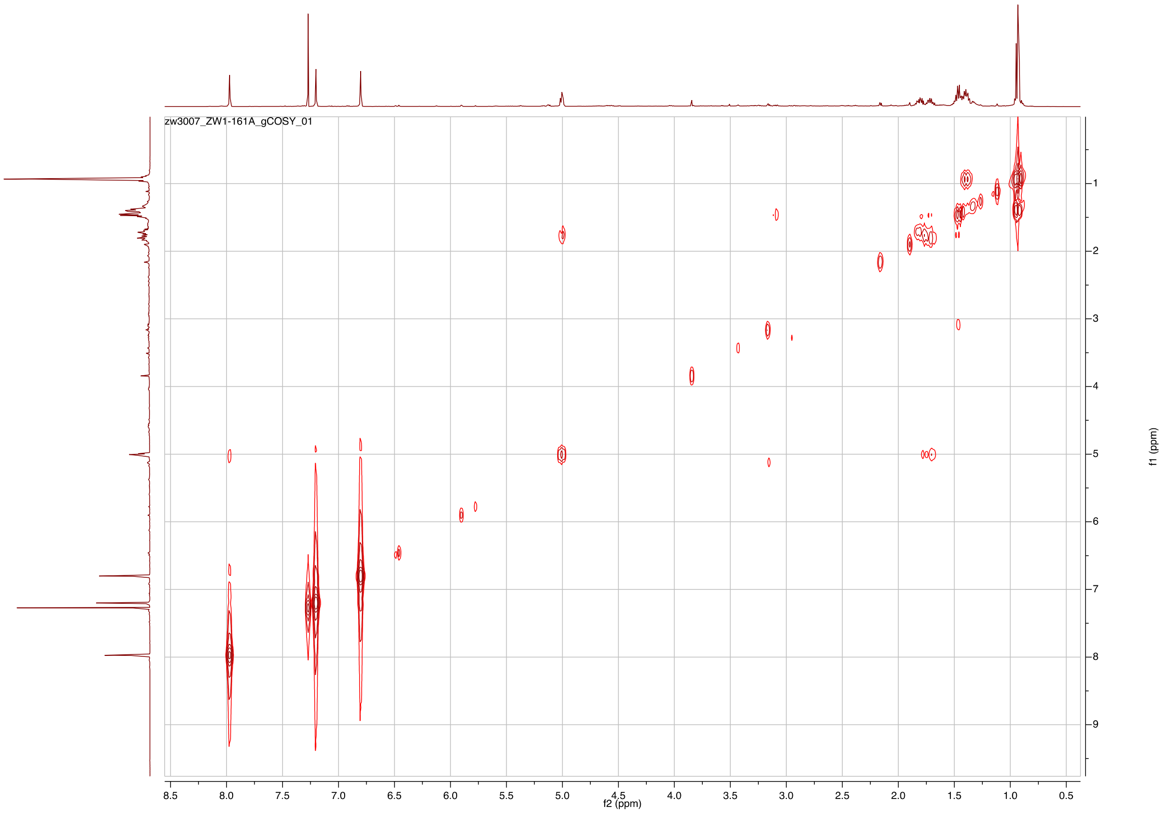

Figure S8. COSY of **4**.

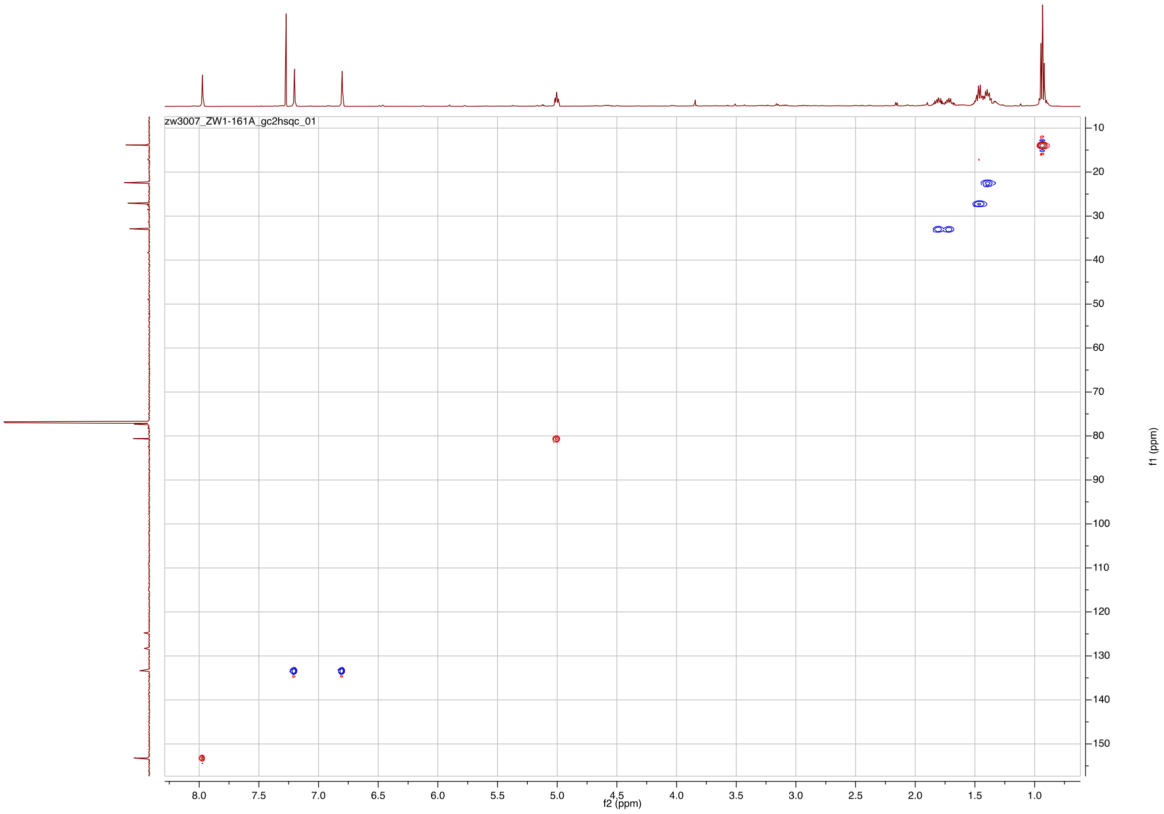

Figure S9. HSQC of **4**

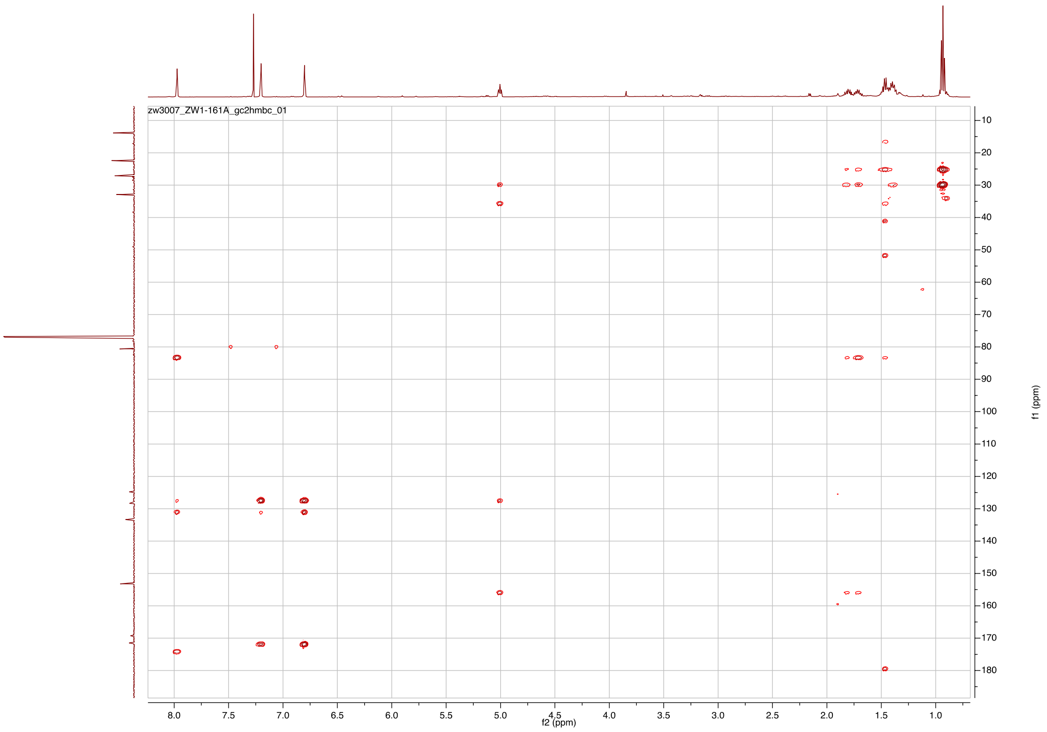

Figure S10. HMBC of **4**.

2. Bioinformatic Analysis

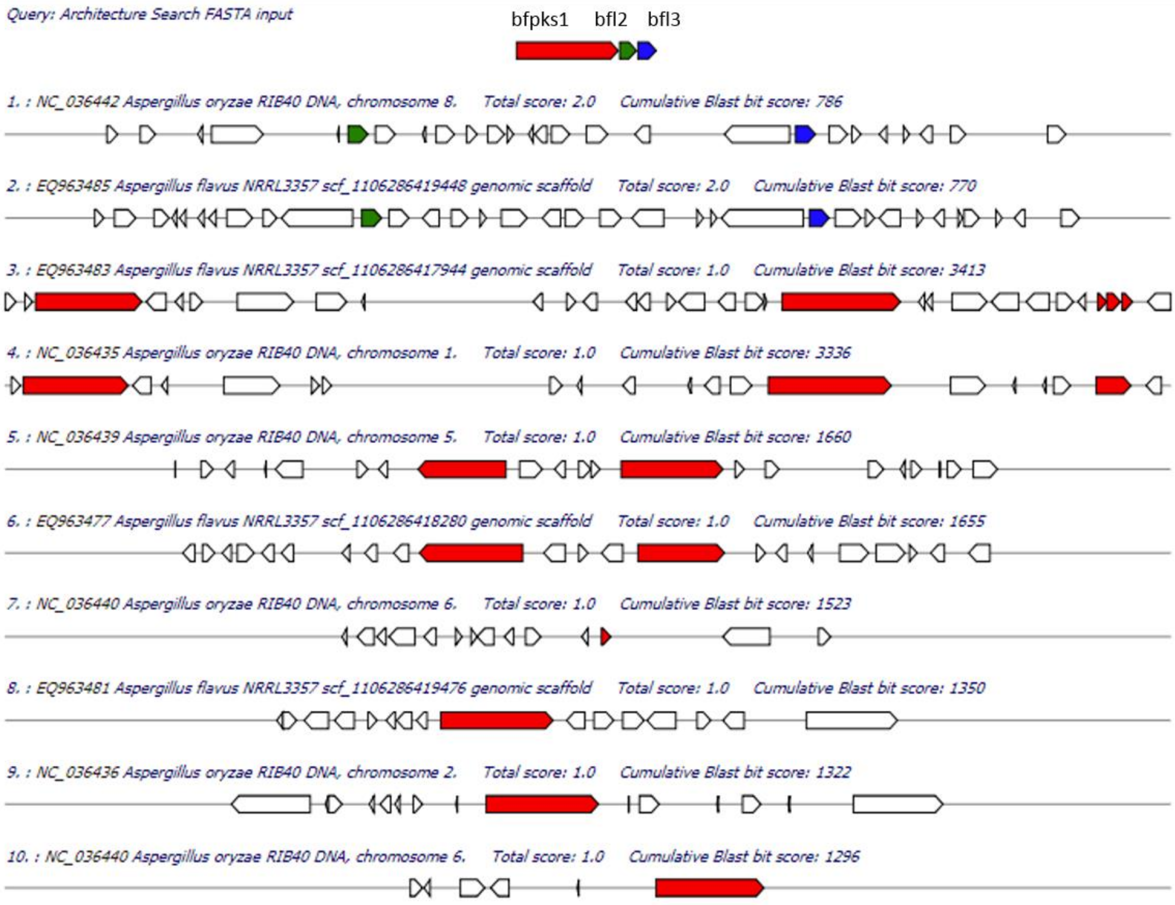

Figure S11. MultiGeneBlast (architecture search) of the byssochlamic acid biosynthetic enzymes bfpks1, bfl2 and bfl3 against the genomes of *Aspergillus oryzae* RIB40 and *A. flavus* NRRL3357. The top two hits are the only gene cluster containing a citrate synthase (bfl2) and 2-methyl citrate dehydratase (bfl3) homologue.

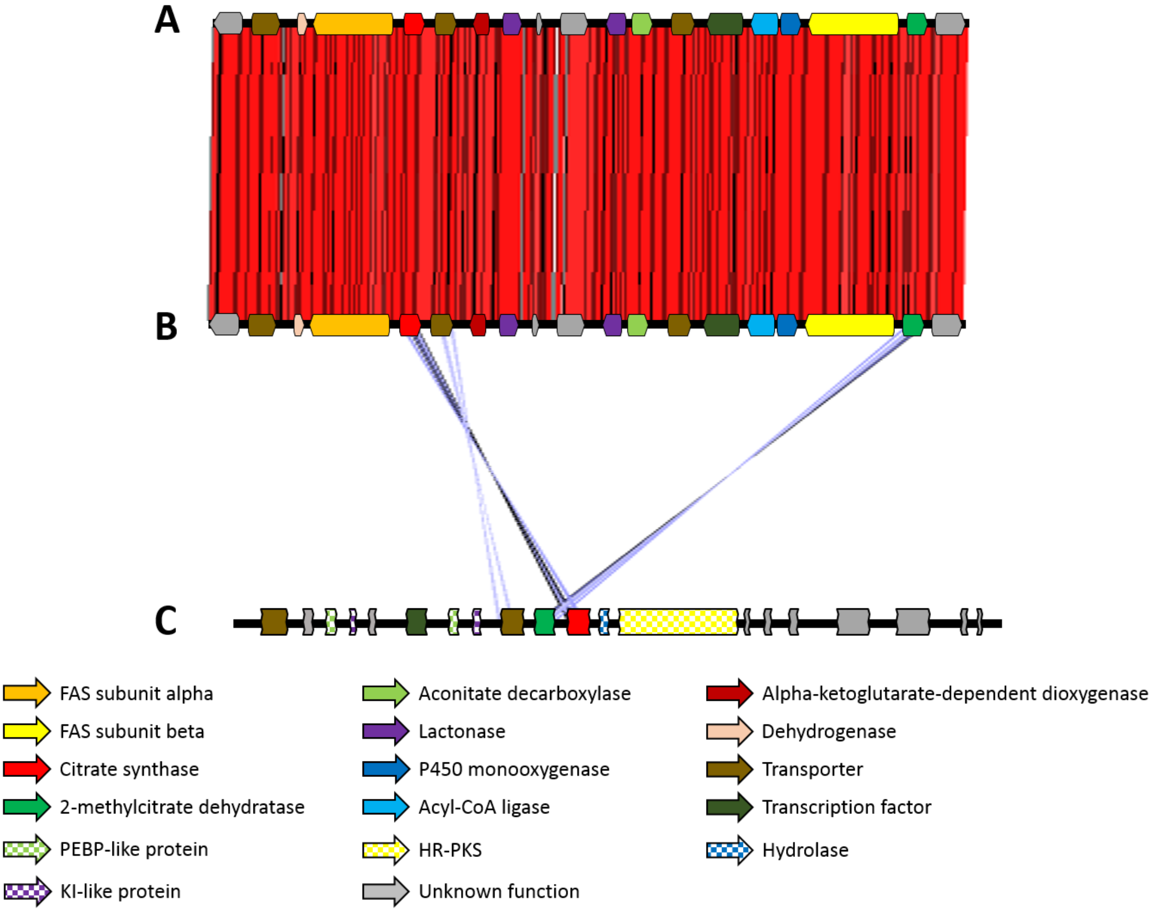

Figure S12. Artemis comparison tool (ACT) analysis between the putative oryzine biosynthetic gene cluster (BGC) of *A. flavus* (A) and *A. oryzae* (B) and the byssochlamic acid BGC of *Byssochlamys fulva* (C). Areas (genes) with high similarity are marked in red (same reading direction) or blue (inverted reading direction).

Table S1. Predicted genes and corresponding nucleotide as well as protein sequences of the re-annotated oryzine BGC from *A. oryzae* RIB40.

Gene	Predicted nucleotide sequence	Predicted protein sequence
oryC	ATGACCGTGTTAACCAGGGAAAAAAAGCCTTATTTTGGCTTGACTGGA<br>GGATGGCTCACATTCTGGGTTACGGTATGGCAATTTTAGCACCTTCGTC<br>GATCACGATGGGCTGACAATTCATTAGGTTGCCTGCGCAACAGATATG<br>TCCTTGTTCGGTTACGACCAGGGTGTCTTCAGTGAGACTACCTAGAATA<br>AATCATGGATCTTTCTAACACACCGCAGGTGGCGTGGTTATCACACGA<br>GACTTTCTGGAAGTCCATGACCTGGTCGGTCCGGAGAAAACCAAGACG<br>CTTTCCACTGTTACAGCGATATACGATGTAGGCTGTTTCTTCGGTGCAA<br>TCGTTGCCTTCACAATCGGAGAGCAACTAGGACGGAAAAAGGCGATC<br>CTGCTAGGAACTACGATTATGGCTATCGGTGCTGTACTGCAGGCTGCCT<br>CTTTCAGTTTGGCACAGATGTTTGTGGGCCGCATTATTCTGGGGTGAGT<br>GAACAGCGTTAACTGTTCTATTAACTTGTCTTGGTTCCGTTGCATTGATG<br>ATGTGTTCAACGATAGTATCGGCAATGGAATCAACACTGCGACTGCAC<br>CTATCTGGCAAACCGAGACATCGCAGCTGAAATGGCGTGGTAAACTGG<br>TCATCTTTGAGATGATGATGAATATCTTCGGCTTCTGCCTCGTCAATTGG<br>ATCAATTATGGTCTCTCCTTCGTCGGTGGTTCCATCGCTTGGCGCTTCCC<br>GCTAGCATTCCAATTCTTCTTCTTGATTATTCTGTGGTCCACTACACCAT<br>GGCTTCCTGAGTCCCCTCGGTAGGCAGTTTGAGCTTCTTTCTCATTACTA<br>AACCTGCTAATGACATGCAGATGGCTCATAGCCCACGGGAGACAAGA<br>AGAAGCCACAGTAGTACTCAGCTGCCTCGAGGCAAAACCCATCGATG<br>ACCCCTTCGTCATCGCGCAACGGAACGAGATCGAGTTTAGCGTGCGAT<br>ACGAACGCGAGAATTCCATGCGGTGGCGAGACCTCTGTCAGAAAAAG<br>GGGAATGACAGCAAGACCCTACGCAGACTTCTCCTCGGTGCCGGCAGT	MTVLTREKKPYFGLTGGWLTFWVTVACATDM<br>SLFGYDQGVFSGVVITRDFLEVHDLVGPEKTKT<br>LSTVTAIYDVGCFFGAIVAFTIGEQLGRKKAILL<br>GTTIMAIGAVLQAASFSLAQMFVGRIILGIGNGI<br>NTATAPIWQTETSQLKWRGKLVIFEMMMNIFG<br>FCLVNWINYGLSFVGGSIAWRFPLAFQFFFLIIL<br>WSTTPWLPESPRWLIAHGRQEEATVVLSCLEAK<br>PIDDPFVIAQRNEIEFSVRYERENSMRWRDLCQ<br>KKGNDSKTLRRLLLGAGSQFMQQFGGINIMSY<br>YLPTVLMDSVGLSDTMARLLAACNALSYLVFS<br>GLAVLLVERIGRRGLMLLSTFGQFLCFLIITILLRF<br>SRISDNGEKFASASVAFFFLYYGAFGIGMLGVP<br>WLYPTEINSLPMRTKGAAVATATDWITNFVVV<br>EITPIGIKNIDWKFWIVWTVTNAAFLPILYFLYPE<br>TANRSLEDMDEYYRSNPALVVTKDPDAICRRRP<br>QKYLQREEEEIERAAAAVDKRALSVGAVEH<br>AEWTNAMGNKS

	CAATTCATGCAGCAATTCGGTGGAATCAATATCATGTCATATTATCTGC<br>CAACGGTGCTTATGGATGTGAGTAACTCTCCCATTTCATCCGATCCACT<br>TTGTACAACAATCTAACGCATGATTGAACCCAGTCCGTCGGGCTTTCCG<br>ATACAATGGCCCGTCTGCTAGCTGCATGTAACGCCCTATCCTACCTTGT<br>ATTCTCCGGCCTCGCAGTCCTCCTAGTCGAGAGAATAGGTCGCCGAGG<br>CTTGATGCTCCTCTCTACTTTCGGCCAGTTCCTCTGTTTCCTCATCATTAC<br>CATCCTACTTCGGTTCTCTCGTATAAGTGACAACGGCGAAAAGTTTGCC<br>TCTGCATCAGTTGCATTCTTCTTCCTCTACTACGGCGCATTTGGCATCGG<br>TATGCTCGGTGTACCTTGGCTATATCCCACGGAGATCAATTCCCTTCCC<br>ATGAGGACCAAGGGAGCGGCTGTTGCGACTGCCACTGATTGGTTTGTT<br>TCTCACTCCGTATTCCCCAGTATACTACTAACTCGGGATGGAAAGGAT<br>AACAAATTTCGTCGTCGTGGAAATAACACCCATCGGAATCAAGAATAT<br>AGACTGGAAATTCTGGATCGTCTGGACGGTCACTAATGCTGCGTTTCTA<br>CCGATTCTTTACTTCCTTTATCCTGAAACAGGTTTGTCTGGCTTCTCGTT<br>GTGGGAAGATGTATTTGCTGATGGTTATGTGTAGCGAACCGAAGCCTT<br>GAGGATATGGATGAGTATTATCGGTCTAATCCGGCGTTGGTAGTCACC<br>AAAGATCCTGATGCTATCTGCCGACGGCGTCCACAGAAGTATCTTCAG<br>AGGGAAGAGGAAGAGATTGAGAGGGCTGCTGCTGCGGTGGATAAGAG<br>GGCTTTGTCGGTTGGAGCTGTGGAGCATGCGGAGTGGACCAACGCTAT<br>GGGGAATAAGAGTTGA	
oryD	ATGCCCGTGGGTACCGAGGCAATCCGCAAAGTCGATTTTCTGGCTGGC<br>ACGGGAAACCGATTCGTGGCTGAGGGTAAGCGGCAGCTGTTTGGTGAA<br>GTTGGCATTGACCTTTTTGCTGGTCCCACCGAGAGCCTCGTCCTGGCAG<br>ATGAGACGGCCGATCCGTTCACGGTGGCTACGGACTTGATCTCTCAGG<br>CTGGGCATGGGCCGGATACACCCGCTGTGTTGATTACGACATGTCCCA<br>AGGTTGGGAGGGAGACGATTGAGATTGTCAATAAGCTGCTCTCTGCGA<br>CGGATCTGTCAACCCCAGATGTGGCGAAGGTATCGTGGGATGCGTTTG<br>GGGAGGTCATTATTGTGGATACTCTGAAGGAGCTTTGGGAGCTGGGTG<br>ATCATTATGCTAGTGAACAGGTTCAGGTGTTTACTAAGGATCCGAGAG<br>ATGCGTTGGACAAGATGTCCAATTATGGGGCGCTATTTCTTGGGGAGA<br>ACACATGCGTTTCATACGGTGACAAGGTAGGTGATTAGAATGAAGAA<br>ACAAACCAGATAGGGGTAGGCCTACTGACTATTCTAAGGTAATTGGAA<br>AAAACCATGTCCTCCTTACTCGGACAACAGCTCGATACACCGGTGGTC<br>TGTGGGTCGGCAAGTATCTCAAAACATGCACCTACCAAGAGGTGACTT<br>CGCCAGAGTCCAGTGGAAAGCTCGGACGCTTGTGTGGACGAGCTGCTC<br>GACCCGAGAGGTTTGAAGCGCATGCGCGATCTGGTGATCTACAAGCTA<br>ACAGGCACATGTGA	MPVGTEAIRKVDFLAGTGNRFVAEGKRQLFGE<br>VGIDLFAGPTESLVLADETADPFTVATDLISQAG<br>HGPDTPAVLITTCPKVGRETIEIVNKLLSATDLST<br>PDVAKVSWDAFGEVIIVDTLKELWELGDHYAS<br>EQVQVFTKDPRDALDKMSNYGALFLGENTCVS<br>YGDKVIGKNHVLLTRTTARYTGGLWVGKYLKT<br>CTYQEVTSPESSGKLGRLCGRAARPERFEAHAR<br>SGDLQANRHM
oryfas A	ATGGTTCAACGCAAAGATCCACCGTCAGAGCAGCTTAGAGCCTACACA<br>TTGCTCATTGAGCTCTTGTCGTACGTTGCAAACCCCATGAAACGTGTGA<br>GATGCTAACGCAGTTTAGGTATCAATTTGCCTTTCCCGTCCGATGGTGA<br>GACTTCCTCGTTACCAACATAACAGAAAAGGGCTAATTAGGAATGCCA<br>GGATTGAAACACAAAATGATCTAATCCAGCGAAATAATACTATCCAGC<br>GCTTCGTTGAAGTGGGACCATCCAATGTCCTGGCGAACATGGCGAAGA<br>AGACAGCCAAGGGCCAATACGCTGAAGAGGATCTGGTGCGCTGCGTA<br>GACCGTCAGTACCTATCGCACGCCGACGACGCCCAGCACATCTACTAC<br>CAGTATGATGAAGAGGCACCGGTGGAAAGCGCTGATAATGAACCGGC<br>CCAACCGGCCGCCTCGTCCACCCCTGCTGCTCCGGCACCAGTGGCTGC<br>GCCACCGGTGGTAGTTCAAACAGCGCCCCAGCCAGCTGCCCAGGCAG<br>CTGTCGCTGTACCCGATGTCGACTTGAGTGCAATCGACGTTGTCATCTC<br>CATAGTCGCTCAGAAGATCCGCAAAGCATTTGACGAGGTACCCGCAGC<br>GAAGTCTATCCGCGATTTATCCGCAGGTACGTTCCGTCTCGATAGCTGC<br>TCATTGTGTGTTCATTCTAACTCGGACAGGCAAATCCACATTACAGAAT<br>GAGCTAATCGGACAGCTGGACGCTGAGTTTCGCGGTCTGCCAGAAGGA<br>AGTGAAGATCTCGCGCTAGAAGCACTGGCCTCCCACTTCACAAACTTC<br>TCTGGACGACCTGGTAAGGTGATGGGCGGACATATCGATCGCCTTGTG<br>GCCGCCCGTATGCCTGCAGGTTTCAACCAAGCCAAGATCAGAGACTAT<br>CTCTCCTCACACTGGGGACTAGGCCTCAATCGGCAGACCACCGTTCTGT<br>GCTATGCGGTAACAATGGAACCGGCTGCTCGCCTCGCCGATGCCGGCC<br>AGGCAACGCAGTTCCTAGATTCAGTGGTGAGCCGATACGGAGGAAAG<br>GCAGGCATAGCTCTACAGAAGAGAGCGGAGGGTGGAGCGTCACAGAC<br>ATCTGCTGTGGCCCAGGTCGACTTGGCTAGTCTTGAAACCCTCAAGAA<br>GGAACAAAATGAGTACTTACACAAACAGTTCCAGCTTCTCGCCAAGCA<br>TCTGGATTTGGATGGCGTGGCGCAACCCAGTCAAACCCAGGTGCAAGG<br>GGAGGATACTGACAGGCTGGCTGAATGGGACGCCGAATTCGATGAAG<br>AGTTTCTCACGGGTATGCGGACGATCTTCGACCCGCGGAAGGCTCGTC<br>GTTATGATTCATGGTGGAATACGGCCCGAGAGGACCTCATGGCCTTGTT<br>GCACGACATCCGTCCTGCTGCCGAGGACAAGGCCTCGCAGCGATACC<br>AGAGCCTTGTCAATCGGTGGTCACCAGAATTGGAGCAGATGCTGGAAC<br>ACAGTGCTCAGGATGACACGACGAAGGAGAAGGCTCAGATGCTGCTT<br>GACGATGTTCGTGCTTCGGGAGTAGCCAACGGGCCAGTCTTTCGTTATA<br>CTCAGCCTGCTATGGCGCCTGAGACGAAAGTCGATGCGAACGGGCGC<br>ATCCAGTACTCTGAGGTGCCTCGTCGTCAGCTCCACGGTGAGAACAAA<br>GCTTCCACCCTGAACTACGCGCAGGTAGTTGCAGCTCGACACCGCGAC<br>GTGCCCTATGCGCACCTCCGCAGCCGAGCCGGTGTGGATTGGAAGTAT<br>GACGACCAGCTCACGGACATGTTTTTGAATATCCTATCTACCGGAGCCT	MVQRKDPPSEQLRAYTLLIELLSYQFAFPVRWIE<br>TQNDLIQRNNTIQRFVEVGPSNVLANMAKKTA<br>KGQYAEEDLVRCVDRQYLSHADDAQHIYYQY<br>DEEAPVESADNEPAQPAASSTPAAPAPVAAPP<br>VVVQTAPQPAAQAAVAVPDVDLSAIDVVISIVA<br>QKIRKAFDEVPAAKSIRDLSAGKSTLQNELIGQL<br>DAEFRGLPEGSEDLALEALASHFTNFSGRPGKV<br>MGGHIDRLVAARMPAGFNQAKIRDYLSSHWG<br>LGLNRQTTVLCYAVTMEPAARLADAGQATQF<br>LDSVVSRYGGKAGIALQKRAEGGASQTSAVAQ<br>VDLASLETLKKEQNEYLHKQFQLLAKHLDLDG<br>VAQPSQTQVQGEDTDRLAEWDAEFDEEFLTGM<br>RTIFDPRKARRYDSWWNTAREDLMALLHDIRP<br>AAEDKASQRYQSLVNRWSPELEQMLEHSAQD<br>DTTKEKAQMLLDDVRASGVANGPVFRYTQPA<br>MAPETKVDANGRIQYSEVPRRQLHGENKASTL<br>NYAQVVAARHRDVPYAHLRSRAGVDWKYDD<br>QLTDMFLNILSTGASTGLSFTGRRVLVTGAGVG<br>SIGADIVAGLLAGGAHVIVTTSRQPSDVAASFR<br>QLYAKVGAPGSELIVLPFNQASKRDCEELINHIY<br>DEQSGYGWDLDFIIPFAAISEIGRQIDKIDSKSEL<br>AHRAMLVNLLRLLGFIKQQKEKRGFDCRPTGV<br>LLPLSPNHGNFGGDGLYSESKLGLETLFNRFHSE<br>GWSDFLCIIGAVIGWTRGTGLMSANNIVAQGM<br>EDSLDILTFSAPEMAFNILSLLSGDILEVADDEPI<br>YADLSGGLQGVSDLKDKISAIRKKIVSDSRIRQA<br>LVAENLHEQKVLRGTKPAEGNVQPPLKRRSNIE<br>PAFPPLSDYNSVTAGLQSLKGMVDLSRTVVVVG<br>YSELGPWGSSRTRWEMEHEGRLSLEGYTELAW<br>MMGLIKHFDGDLKGKPYTGWVDSKTKEAVDE<br>ADIEEKYGQHILGHAGIRVIEPELSEGYDPSQKEI<br>MHEVVIDEDLPPFEAPQGVAQAFKLRHGDKVI<br>LTPIEGSESVKVVVKSGAVFMVPKAMAFNRFVA<br>GQLPSGWDPTRYGIPEDIVAQVDPMTVYVLCC<br>VSEAMYSAGLEDPFELYRHIHVSELANCVGTGA<br>GGLLAMRGVYRDRYLDRPVQSDILQESFLNAM<br>NAWTNMLLMGAAGPIKSPSGTCATSVESMDIA<br>CEAIQTLKAKVAIVGGSDDFQEEMSYEFGNMK

	CTACCGGACTGTCATTTACCGGCAGAAGAGTACTGGTGACCGGTGCTG<br>GAGTCGGTTCGATTGGAGCAGACATCGTGGCCGGGTTGCTGGCCGGTG<br>GAGCGCACGTTATCGTCACCACCAGTCGTCAGCCTTCAGATGTCGCTG<br>CATCCTTCCGCCAGCTCTATGCGAAGGTCGGAGCTCCTGGATCAGAAT<br>TGATTGTGCTGCCATTCAACCAAGCCAGCAAGCGTGATTGCGAGGAAT<br>TAATCAACCACATCTACGACGAGCAGTCGGGTTATGGTTGGGACCTCG<br>ACTTTATCATCCCCTTCGCTGCCATCTCCGAGATCGGACGGCAGATTGA<br>CAAAATCGACTCCAAGTCGGAATTGGCGCACCGTGCCATGCTGGTTAA<br>TCTGCTCCGCTTGTTGGGATTCATCAAACAGCAGAAGGAAAAACGGGG<br>TTTTGATTGTCGGCCCACAGGTGTCTTGCTCCCCTTGTCTCCAAACCATG<br>GTAACTTCGGTGGAGACGGCTTGTATTCTGAGTCCAAGCTGGGGCTGG<br>AGACCCTGTTCAATCGCTTCCATTCTGAGGGCTGGTCTGACTTCCTTTGC<br>ATTATCGGTGCCGTGATCGGCTGGACCCGCGGCACGGGCCTGATGAGT<br>GCCAACAATATCGTCGCCCAGGGTATGGAAGACTCATTGGATATACTG<br>ACATTTTCTGCCCCCGAGATGGCTTTCAATATTCTGTCTCTCCTGTCAGG<br>CGACATCCTCGAAGTGGCGGACGATGAACCCATTTATGCGGATTTGAG<br>TGGAGGCCTTCAAGGTGTCAGTGACCTCAAGGACAAGATTTCGGCTAT<br>TCGCAAGAAGATCGTCTCGGATAGCCGGATCCGCCAGGCGCTGGTCGC<br>CGAGAATCTACACGAGCAAAAGGTTCTCCGAGGCACGAAGCCTGCGG<br>AGGGTAATGTTCAACCTCCTCTTAAACGCCGCTCCAACATTGAGCCGG<br>CCTTCCCACCTCTTTCAGATTACAACTCGGTGACAGCTGGCCTCCAGTC<br>CTTGAAGGGAATGGTGGACCTGTCTCGCACGGTGGTGGTGGTAGGTTA<br>CTCGGAGCTGGGCCCTTGGGGCTCTTCGCGTACACGCTGGGAGATGGA<br>GCATGAGGGCCGCTTGTCTCTGGAAGGTTATACCGAACTGGCATGGAT<br>GATGGGCCTTATTAAGCACTTTGACGGTGATCTTAAAGGCAAACCATA<br>CACCGGCTGGGTTGATTCCAAGACCAAAGAAGCGGTGGACGAGGCGG<br>ATATTGAGGAGAAATATGGGCAGCATATCTTGGGCCATGCTGGCATTC<br>GGGTGATAGAGCCCGAGCTGTCGGAGGGATATGACCCTTCCCAGAAA<br>GAGATCATGCACGAAGTGGTTATCGATGAGGACCTCCCTCCCTTTGAA<br>GCTCCCCAGGGTGTCGCCCAGGCATTTAAGCTGCGACATGGTGATAAG<br>GTCATCTTGACGCCTATTGAAGGTTCAGAATCTGTCAAGGTTGTCGTCA<br>AGAGTGGAGCTGTCTTCATGGTGCCTAAAGCTATGGCATTCAATCGGTT<br>TGTCGCCGGTCAGCTCCCGTCCGGGTGGGATCCAACTCGTTACGGTATT<br>CCTGAGGACATTGTCGCTCAGGTGGATCCTATGACTGTCTATGTTCTAT<br>GCTGTGTATCTGAGGCTATGTATAGTGCTGGACTAGAGGATCCATTTGA<br>GCTGTACCGCCACATCCACGTATCTGAATTGGCTAACTGCGTCGGCACT<br>GGTGCCGGAGGCTTGTTGGCTATGCGTGGCGTGTATCGGGACAGATAT<br>CTTGATCGACCTGTGCAAAGCGACATTCTCCAGGAGTCTTTCCTCAACG<br>CCATGAACGCCTGGACGAATATGCTTTTAATGGGGGCTGCGGGTCCTA<br>TCAAGTCGCCCTCAGGCACATGTGCGACCTCGGTAGAGTCTATGGATA<br>TAGCCTGTGAAGCAATCCAGACACTTAAGGCCAAGGTTGCTATCGTTG<br>GTGGCTCCGATGACTTCCAGGAAGAGATGTCCTATGAGTTCGGTAACA<br>TGAAGGCCACTGCCAACGCAGAGGACGAGTTGGAGAAGGGATATCTT<br>CCTTCAGAAATGTCCCGACCTACAGCATCCTCTCGCAGTGGCTTCGTCG<br>AGTCCGCTGGCTGTGGTATCCAACTGGTCATGAGCGCCGAGCTAGCCC<br>TTCAGATGGGCCTTCCCATCTACGGAATAGTCGCTTACTCCCAAATGGC<br>AGGCGATAAGGTCGGCCGGTCTGTGCCTGCACCAGGCCAGGGTGTACT<br>CACGGCCGCCCGGGAAAGCATTGATGCGGCGCAATCACCCTTACTGGA<br>TGTGCAATACCGCAAGGCACGATTGGATGAAGCTGTCTCTGAGATCAA<br>GAGATGGCGACACAAAGAGTCACAGAAGCTCATCGCGTCCACGACCT<br>CTAAAGAGTTCAAAGATCTCGATGCGCACCTGCAACACATCAACAAC<br>ATCGCCGCGACCCGCATCCGGGATGCTCAATGGACATGGAATAATAAC<br>ATCCGTCATATAGATCCCACTATTGCTCCCATGCGCGCCGCCCTGGCAA<br>CGTGGGGGTTGAGTGTCGACGACATTCAAGTGGCGTCCTTCCACGGCA<br>CGTCAACCAAGGCCAATGACAAGAATGAAAGCAACGTTATCAACCAG<br>CAAATGACTCACCTGTCGCGCACGGTTGGCAACCCACTTCTCGTCATAT<br>GCCAGAAATCCCTAACAGGCCACCCGAAGGGTGCAGCCGGAGCGTGG<br>ATGTTCAATGGCTGTCTCCAGGCTCTCCAAACCGGAATTGTACCCGGC<br>AACAGGAATGCAGATAATGTCGACGTGGCGCTCCAGCAGTTCAAACA<br>TCTGGTTTACCCATCACAGACGATCCATACATCAGGTATCAAGGCATTC<br>ATGTTGACATCGTTCGGCTTCGGACAGAAGGGTGGCCTCGTTGTTGGTA<br>TCGCCCCTCGCTATCTCTTCTCCACTATTACGGCCAATAAGTTTGAAGA<br>TTACCGCGAGCGGGTGCTTCAACGCCAGCAAAAGATTATTCCCGTGTT<br>CCAAAGGCGTATGGCACAGGGCCGTCTTTTCCAGATCAAAGATCAGTC<br>GGCTTGGACAAGTGATCAAGAAAAGGACGTCTTTCTCAATCCCCAGGC<br>CCGTGTCGCTCAGAAGTCTACTGGCGAATACAGCTTTCCAACGACGGT<br>TGCCCCAGTTGCATCATCACTGCCCGCGAGAACTGTTTCCGACGACAA<br>GCAGCTCTTCGCTAGATCTTCTGATCAATGGCTGCGCGACAGCATAAGT<br>AAGGAGCAGGGAAATGTGTCCGTTGGTGTAGACATTGAGTCTATCTCC<br>AGTGTCAATATCGAGGATGAAATATTCCTGGAACGCAACTTCACGCCA<br>GGGGAGCTCAAATACTGTCAAGGTAGCCCAGATAAGCAAGCAAGCCT<br>TTCAGGGCGCTGGGCGGCCAAGGAAGCGATCTTCAAGAGTCTACAGAT<br>ACCGTCTGAAGGTGCAGGCGCTGCGATGCGGGATATTGAGATTGTCAG<br>CAATGGTGCTCAGCCACCAACCGTTCTGGTAAGACCGAGCCCACTAGT<br>CCTCTCGTTATAGCTGACCATTGCTTAGCTTCACAATCGGGCCAAATCG	ATANAEDELEKGYLPSEMSRPTASSRSGFVESA<br>GCGIQLVMSAELALQMGLPIYGIVAYSQMAGD<br>KVGRSVPAPGQGVLTAARESIDAAQSPLLDVQY<br>RKARLDEAVSEIKRWRHKESQKLIASTTSKEFKD<br>LDAHLQHINNIAATRIRDAQWTWNNNIRHIDP<br>TIAPMRAALATWGLSVDDIQVASFHGTSTKAN<br>DKNESNVINQQMTHLSRTVGNPLLVICQKSLTG<br>HPKGAAGAWMFNGCLQALQTGIVPGNRNAD<br>NVDVALQQFKHLVYPSQTIHTSGIKAFMLTSFG<br>FGQKGGLVVGIAPRYLFSTITANKFEDYRERVLQ<br>RQQKIIPVFQRRMAQGRLFQIKDQSAWTSDQEK<br>DVFLNPQARVAQKSTGEYSFPTTVAPVASSLPA<br>RTVSDDKQLFARSSDQWLRDSISKEQGNVSVGV<br>DIESISSVNIEDEIFLERNFTPGELKYCQGSPDKQ<br>ASLSGRWAAKEAIFKSLQIPSEGAGAAMRDIEIV<br>SNGAQPPTVLLHNRAKSAADAQKVEEVQVSIT<br>HSPESAMAIALARRRL

	GCTGCCGATGCCCAAAAGGTGGAAGAGGTCCAGGTTAGTATCACGCA<br>CTCCCCAGAGAGTGCCATGGCTATTGCTTTAGCGCGACGTCGTTTATAG	
oryE	ATGACTGTGACTCAAGAAGCCTCTCCCAAGCGGGAATCCCTACACATT<br>ATTGATGACCGTACTGGATCTTATTACTCGATTCCCATTGTGAACAATG<br>CCATCAATGCCAGCGATTTCAAAAAAGTGACCGCGCCAGAAGACAAA<br>GCTTACCCTGCCAACCAAACGGAGAATGGGCTGCGGGTTTACGACCCA<br>GGCTACTCCAACACTGCCGTCAGTCACAGTAAGATCACCTATATTGAT<br>GGGCTCAAGGGCACTATCCAGTACCGTGGGTACTCCATCAATGACATT<br>GTCGGACGCAAGACGTTTATCGATACCGCGCATCTCCTGATCTGGGGT<br>CACTGGCCCTCCACCGCCGAGGCCGAAACCCTTCAGCAACGCCTCGAC<br>CAGGTTCCTGTCCCTCAGGATTTTGTCTTCAATGTGATCAAATCCTTTCC<br>GTACGTCTTCCCAAAACTGACCCTCCTACGGGTGCTTCTTGCTGACCAG<br>CAGCAGACGAGATGGTTCCCTTATGGGAATGGTAATCGCCGGTCTATC<br>AGCGCTCCAGTCCAGTGATATGAATGCGATCCCGGCTCATGTGGGAAA<br>GACCATCTATCTGAACAATCCCGAGCTAGCCGATCAACAGATCATTCG<br>GGTCATGGCAAACATGTCCATGTTGACAGCCGCGGCATACTGCCATCA<br>CATCGGACGTGACTTCACTCCACCCCGCGCGGGGCTATCGTACATTGA<br>GAACTTCCTACTCATGACTGGTCATGTTGAGGCAGCTACTGGCCTGCCA<br>AACCCGCGATACGTGAATGCCATTGAGCGACTCTGGGTCCTCATTGCC<br>GATCATGAAATGACCTGCTCCACTGCGGCCCTTCTTCAAACCGCATCTG<br>CCCTACCAGATGTTATCTCCTGCATGGTCTCCGCCATCTCGGCATTGTA<br>TGGTCCTCTCCATGGTGGAGCCATTGAAGTCGCTTACAAGAATATTGA<br>GAGCATCGGCTCCATCTCCAATGTACCTGCCAAGATCGCCCGCGTCAA<br>AGCAGGAAAGGAACGTCTGTATGGTTACGGCCACCGAGTCTACCGTGT<br>GACAGATCCTCGATTTGTCTTCATCCGTGAGATCCTCAATGAACTGTCC<br>GAGGAAGTGGAGAAGGACCCCTTGTTGAAAGTTGCATTCGAGGTCGAC<br>CGTGTTGCCTCAGAAGATGAGTACTTCACGTCCAGGAATCTCCGCCCC<br>AACGCGGATTTGTTCGCTGCGTTTGTCTACAAGGCTCTGTATGTTCCTTT<br>TCCCGTGATTTCTGAATTATAGTGGTATTAACCCTGTGCTATAGTGGGTT<br>CCCGCCCCGAATTCATCCTGCCTCTGTCGATTCTCTCCCGCACACAAGGG<br>TTTATGGCCCACTGGAGAGAGGCCATGGGTATGCCAGAATCTACCCTA<br>AATTCCTCTAGAAGCATCTGTCCTGACACGAATATAGGCAATCCTCCTC<br>GAATCTGGCGGCCGGGTCAGATCTATACCGGTGACCTGAACAAGTCCA<br>TGGATGAGTGA	MTVTQEASPKRESLHIIDDRTGSYYSIPIVNNAIN<br>ASDFKKVTAPEDKAYPANQTENGLRVYDPGYS<br>NTAVSHSKITYIDGLKGTIQYRGYSINDIVGRKTF<br>IDTAHLLIWGHWPSTAEAETLQQRLDQVPVPQ<br>DFVFNVIKSFPRDGSLMGMVIAGLSALQSSDMN<br>AIPAHVGKTIYLNNPELADQQIIRVMANMSML<br>TAAAYCHHIGRDFTPPRAGLSYIENFLLMTGHV<br>EAATGLPNPRYVNAIERLWVLIADHEMTCSTA<br>ALLQTASALPDVISCMVSAISALYGPLHGGAIEV<br>AYKNIESIGSISNVPAKIARVKAGKERLYGYGHR<br>VYRVTDPRFVFIREILNELSEEVEKDPLLKVAFEV<br>DRVASEDEYFTSRNLRPNADLFAAFVYKALGFP<br>PEFILPLSILSRTQGFMAHWREAMGNPPRIWRP<br>GQIYTGDLNKSMDE
oryF	ATGGCCGAAGAAGTAAACGAAAGAACGAGACTGCTTTCCCAGTCCGA<br>TGTAAGTTTGTCGCTCGGCTTCTGGCCGTGTCTAAAGCTAATGCCCCAA<br>GGACCCTTCTCCTTCTCTAGAAGAGCTAGAAGAGTGGGAAGAGCCTCG<br>CAATTGGAAGGTTTCCTACCGCTGGCTATGTATTGCCGTGATTTCAGTTT<br>ACGGACTGATCAGTCCAGTCATCGCAGCAATCATCGTGCCTGCCATGC<br>CGCAAATAGCCACAGACTTGAATGTTACCGACCCAGGAATGCTGCAA<br>GCCTTCGTTTCTGTTTATGTCCTCGGCTGGAGCTTTGCACCGCTGGTGGT<br>CGGTCCTTTGTCCGAAGTCTACGGGCGCATCTCGTTGTTGAATACCGGG<br>CATGGCTTGTTTCTTGTCTTTAATGCCTTATGCGCGTTCGCTCGTAGTGA<br>CTACGAATTGCTCATTCTTCGGTTTATCACGGGCGCCGTTGGTAGCGCT<br>CCACTCTCTGTGAGTAATATTCAGTCATACATTAACCATCGCTGACGAA<br>GGAAATAGATCGGCGCAGGTATAATCGGTGACCTATGGGCTCCAGAA<br>GAGCGAGGCCTCTCCATCTCCCTTTACACTTTGGGGCCCCTGCTTGGTC<br>CGGCCATCGCACCCATCACAGGTGCATACATCGTGTCCCACACATCAT<br>GGCGGGCAATATTCGCCTGGTGCTCGCTTTACATCCTCATCACATGGGT<br>GGTCGGTCTTTGTACACTTCGCGAGACCTTCCGGCCAGTCCTCATCCAA<br>CGCAAACAAGCTGCTGCCGTCCGCAAAGGTCAGCTGCCGGGCTCTGTA<br>CAGCACCATCATAAGAGTCTAGCAGATGTATTCAGACAGGATCTCCGC<br>CGACCCTTCACCTTCCTCGGAACCCAGCCCATCATACAAGTCTTGTCCC<br>TCTTCATGGGCTATCTGTTTGGCCTCAACCACCTCTCCATCACCACATTC<br>GAGTCTGTCTGGACAGACATCTACAACCAAACTCCATCCCGCGCAGCA<br>CTAAACTACATCTCCATCGCCGGCGGCTTCATCCTCGGCAGCCAAATC<br>ACAGGCTCCCTCAACGACCGGGTACCCTCCCTCATCCCCTTTCTCATCG<br>CATCCAGATTTCTAACAACCCCAGATCTACATCTACTACACCTCCAAA<br>GACCCCGCCAAAGGAACTCCCGAACTTCGAACAATCCTCATGCTCCCA<br>GCCGCCCTGCTCGTTCCTACCGGCCTACTAATCTACGGCTGGTCCGCCC<br>AGACCCACAGCCACTGGATCATGCCCAACATCGGAATCGGCATCTATG<br>CCCTGGGCCTGATAATGAGTTACCAATGTATTCAGGCCTATGTGCTGGA<br>CTGCTATGCGGTGTATGCTGCGTCAGCGATGGGTGCCCTGACGATTCTG<br>CGGTCGCTGCTTGGTTTTGTTTTACCGATTTTGGCTCCCTTGATCTATAG<br>GACGCTTGGGTATGGATGGGGCAGTTCCTTGTTGGCGCTTTGGGCTTTG<br>GTGATGGGTGGGTTGGTTCCGATTTTGCTGTGGCGGTATGGAGCTGTTT<br>TGAGGAAACGGAGTGGGATTCTTGAGGAGATGTAG	MAEEVNERTRLLSQSDDPSPSLEELEEWEEPRN<br>WKVSYRWLCIAVISVYGLISPVIAAIIVPAMPQIA<br>TDLNVTDPGMLQAFVSVYVLGWSFAPLVVGPL<br>SEVYGRISLLNTGHGLFLVFNALCAFARSDYELL<br>ILRFITGAVGSAPLSIGAGIIGDLWAPEERGLSISL<br>YTLGPLLGPAIAPITGAYIVSHTSWRAIFAWCSL<br>YILITWVVGLCTLRETFRPVLIQRKQAAAVRKG<br>QLPGSVQHHHKSLADVFRQDLRRPFTFLGTQPI<br>IQVLSLFMGYLFGLNHLSITTFESVWTDIYNQTP<br>SRAALNYISIAGGFILGSQITGSLNDRIYIYYTSKD<br>PAKGTPELRTILMLPAALLVPTGLLIYGWSAQT<br>HSHWIMPNIGIGIYALGLIMSYQCIQAYVLDCY<br>AVYAASAMGALTILRSLLGFVLPILAPLIYRTLG<br>YGWGSSLLALWALVMGGLVPILLWRYGAVLR<br>KRSGILEEM
oryG	ATGCACTCCACCAAGGTCACCTACCCCGAGCCTATGCAGCTCAGCGGT<br>ATTCTCGATCAATACGAGTCCTTCCAGGTCACTCCCTGCATTGGTACTG<br>AATTCCCCAAGGCGAACTTGGCCGAATGGCTCCACTCGCCCAATGCCG<br>ATGCTCTCTTGCGCGACCTGGCCATTACGAGTAAGTATCATCTTGACCC<br>ACAACAAGAAAAGCCACACAGCAACTAACTGATCACACTAGTTGCGC<br>AACGGGGAGTTGTTTTCTTCCGTGCCCAGACTGACCTGGACGGAGAAC<br>TCCAGAAGGAGCTCACCCACCGCTTGGGTGTGCAATCTGGCAAGCCAG<br>CTGGCCACCGTCTGAGCAAGCACCCCCTCCACCTTATCCGTAAGGATG	MHSTKVTYPEPMQLSGILDQYESFQVTPCIGTEF<br>PKANLAEWLHSPNADALLRDLAITIAQRGVVF<br>FRAQTDLDGELQKELTHRLGVQSGKPAGHRLS<br>KHPLHLIRKDDPEMGVLDPGRQQKLHGVENT<br>QKRQRAVLEYHSDGSYEVCPPDFTMLRMTEIPP<br>TGGDTLWASGYELYDRLSTPYQKFFESLTAQHE<br>VPSLRKLAETEPGIYDGPRGAPANTDMQFKQS<br>HPMVRTHPVTGWKTLFAGGLHCRRVNDVTDF

	ACCCAGAAATGGGCGTCCTCGACCCCGGCCGCCAGCAGAAGCTGCAC<br>GGTGTCGAGAACACCCAGAAGCGTCAGCGCGCCGTCCTGGAGTACCA<br>CAGCGATGGCTCCTATGAAGTCTGCCCCCCTGACTTTACCATGCTCCGC<br>ATGACCGAGATCCCCCCCACCGGTACGCTAACCACTATCAATCTACTG<br>CACCCCACATACTGACGAATCCAGGTGGCGACACCCTCTGGGCTTCCG<br>GATACGAGCTCTATGACCGTCTCTCAACCCCCTACCAGAAATTCTTCGA<br>GTCTCTTACCGCCCAGCACGAGGTTCCCTCCCTGCGCAAGCTGGCTGA<br>AACCGAACCCGGCATCTACGATGGCCCTCGTGGTGCTCCCGCCAACAC<br>CGACATGCAGTTCAAGCAGTCTCACGTACGTAGCCTACCCCCAATCAC<br>AGTCTCTGTTCCCATCTAATCCTCTACAGCCCATGGTCCGCACCCACCC<br>CGTAACAGGCTGGAAGACCCTCTTCGCAGGCGGTCTTCACTGCCGCCG<br>CGTCAACGACGTTACCGATTTCGAGAGCGAACAGCTCCTGAGCAAGAT<br>CATCTCTCTCGTCGGCGACAACCACGACCTCCAAGTCCGCTTCCGCTGG<br>AACAACCCCGGCGATGTTGGTGAGTACAACCTCCAAATCCAAACCATG<br>CCTAACACCCTGTCTAACCTCAATAGCTATCTGGGACAACCGTTGCGTC<br>CTGCACTGCCCTACCCAGGACCACTATGGCCTCGGAGGCCGCATGGGA<br>TATCGCACCATGGGTATCGCTGAGAAGCCTTACTTGGACCCTAACAGC<br>CCTTCTCGCCAGGAGGCTTTGGCAGCTGCCGCTAAGTGA	ESEQLLSKIISLVGDNHDLQVRFRWNNPGDVAI<br>WDNRCVLHCPTQDHYGLGGRMGYRTMGIAE<br>KPYLDPNSPSRQEALAAAAK
oryH	ATGTATTTATCTCTGCGATTGGTCTCCCTGGCGCTATGCATAGCCCCGCT<br>AGCATCTGCCGGGAACAATTCCACCCCCCTGGTTCAAGCCTGCACTGG<br>CTACAGCTTCCCACGTGTCGCCTGCATGTACCGTTATGCGTCGAAAATG<br>CCCCTTGACTTCTACCGCAAGGCCAGCGTCGACATCAGTAACGTGGAC<br>ACTTACTCATCGACCGAAGTGGCCAATGACGACTCGTTCCAGCAGGTT<br>GGGAAGGCCACATTCCTAGTCTGGGATCAACAGCGTGGTAGCGAAATC<br>CTAGGATCCGACCCGGCGTATGATATTGTGTTTACTATCTCAACTGGCG<br>GACACGAGGCGCCCGTATATGTCCCGGACACCAACGAGCTGTGGTTCT<br>CTGAACTGGGTAAAGGAGAGCTACACCAGCAGGTCATCAGTCTGGAT<br>GGTGATTCCCCTACCATCTCAGAGGTTCTGACCGACCCACCTCTCTACG<br>CTCCATCCGGGGCCCGCTATAGGAATGGAAAGATCTATTTCTCCGCTG<br>GAGGTGGCAATTCCACCCTGGAAGGGGGTCCTTATCACCCAGGAATCT<br>ACTCGGTGGATCCCAAAACCCGGAAATCCACCATTGAGGTCAACAACT<br>ATTTTGGATGGTACTTTAACCAGGTAAGCATGCCCACTGCCCATACGC<br>GAGGCGGGTCCCCTTTGTCGCATTTCTGCACATTGGTGGATCCACGCCT<br>AGTGGCTCACGGACTAACTTTTCAACACAGGCCAACGACATGGATATT<br>GACCAACACGGTCGCATCTGGTTTAGTGATCCTTGTAAGTTCCTGGACC<br>CGGGATCTCACCTTAGTCCATACTGATGCAGCTATAGTCCTCGCCCGCA<br>ACCACGGTACCTCGACAGTCGCCCCTCAGGTCCAGGCATCGGTTTACC<br>GTTACGACCCAGAAACTGGAGCAGTGAATATTGTGGATGACACCCTGC<br>ATTGTCCCAATGGTGTTGCTTTCTCTCCCGACTACAAAACCCTCTACCT<br>CACAGACACTGATGCCGGTGTCCCCATGATTGACCCGAGAGTGCCATT<br>GAGCGAGGTGCCATCGCTCCAGTACAACTCCACCAACCGTCGCACAGT<br>CTACGCTTTCGACGTGAGCGAGGATGGATCGTATCTCAAGAACCGCCG<br>ACCGATCTACACAGCGAAGGACTTCGTTCCTGATGGTCTTAAGGTGGC<br>TAGCAATGGATATGTGATTACCGGGGCGGGCAAGGGTGTTGACATCCT<br>TGATACCACCGGCACACCATTGCTAAGCATCCAGACTAACTTCACCGC<br>CGTGAACATGGTCTTTGGTGGTAAGAACTTGGATGAGCTGTGGATTGTT<br>GGGCACGGCGCTGTTGCGCGAGCTAGACTGAACTTGACTGGTCCTGCT<br>CTAGAGTAA	MYLSLRLVSLALCIAPLASAGNNSTPLVQACTG<br>YSFPRVACMYRYASKMPLDFYRKASVDISNVDT<br>YSSTEVANDDSFQQVGKATFLVWDQQRGSEIL<br>GSDPAYDIVFTISTGGHEAPVYVPDTNELWFSEL<br>GKGELHQQVISLDGDSPTISEVLTDPPLYAPSGA<br>RYRNGKIYFSAGGGNSTLEGGPYHPGIYSVDPK<br>TRKSTIEVNNYFGWYFNQANDMDIDQHGRIWF<br>SDPFLARNHGTSTVAPQVQASVYRYDPETGAV<br>NIVDDTLHCPNGVAFSPDYKTLYLTDTDAGVP<br>MIDPRVPLSEVPSLQYNSTNRRTVYAFDVSEDGS<br>YLKNRRPIYTAKDFVPDGLKVASNGYVITGAGK<br>GVDILDTTGTPLLSIQTNFTAVNMVFGGKNLDE<br>LWIVGHGAVARARLNLTGPALE
oryJ	ATGGGCTCCCTTCCTGAGAAAGACTTCCCCCAAGTCCACCGCTTCATCA<br>CGACCCACAAGGAAGACGGAACCCCCACCTTCGAGACCAAAATCCCC<br>GAGCCAATCGAATGGGAGCGCACCAACATTGGCGTCGATTTCTTCCTC<br>GCCTACACCCTCGGGTCCTTTCCCGCTCCCCTCAGCCACGATGCCGACT<br>TGAACCAATACAAGGACCACCTCGTCAATCACCCTCCCTTCATGATCC<br>CCGGCGGTGCCGTCGTGCGCTACGTCGACTACCACCCGGGCTGTGAGC<br>CCATGTGGCATCGCACTGTGACCGTGGACTTCGGAGTGGTCATTGAGG<br>GCGAGCTGGAGCTGGAGGTGGAAGGCGGCGAGAAGCGATTGATGAAG<br>AGGGGTGATGTTGCCGTGCAGAGAGGTACCAACCACTGCTGGCGCAA<br>CCCCAGCAAGACTCAGTTTGCGCGCGCCTTGTATATTGCCCTGGATGCC<br>AAGCCGGTCATCGTCAATGGCCAGGAGTTGGGCGAGTCGTTGGGCGAG<br>GTTAAGCACTGA	MGSLPEKDFPQVHRFITTHKEDGTPTFETKIPEPI<br>EWERTNIGVDFFLAYTLGSFPAPLSHDADLNQY<br>KDHLVNHPPFMIPGGAVVRYVDYHPGCEPMW<br>HRTVTVDFGVVIEGELELEVEGGEKRLMKRGD<br>VAVQRGTNHCWRNPSKTQFARALYIALDAKP<br>VIVNGQELGESLGEVKH
oryK	ATGGATATTTATGAAGCAGCAAGCCAAGGCCGCATCGACGCGATAAA<br>ATTTGCCGTTGAGCAAGGCTGTGATGTCGACGGTCCCAATGAAGACGG<br>GAAGACGCCTCTCTGGTTCGCTGTGCAGAGTGGCCAGCCAGAAGCCTG<br>CAGATTTCTTATGAGTCTAGGAGCTGGCAGGGGGCCGCAAAATCCTAG<br>CCTCCTCGAAGTCGCTGTTGGAGGGGGGTACGCGGATATTGTCGCACT<br>GCTGTGGCCGCACTGCAATGCAGAAAGGGAACATCGTTCTTTAAAAAC<br>TGCCATCTCACTGGGCTTTCATGAAATCGCTGATTTCTTGATAGAGACG<br>GGCGCGTTTGAATACCAGGACTCTGAAGTCAGTGGCACTGAATCCCTC<br>ATAGAAGATGGCTCCCCCGAGAGAGAATCCACAGTTTTTCAGCAATGG<br>GAGCGATTTCTGTTCGTGCGACGTGGGCAGAAGCTGCCTTTGCATCGCG<br>TATTTTTTGACTACGCTCTTCTTCTCGCCACGAAGGCAGGCCGCAATGC<br>AGGGCTTCGACTGGTAGAGTTTCTGCTTGGGGAATCCATGCCCGACGT<br>GAACTGCAAGATCATGATCAATGGGCAGTTTGAGACACCTTTGACGGC<br>GGCCGCTGAGAAGGGTAACCTGGAGATCCTCGCAACTCTGATCGACCA<br>CCCAAACATCGACTTGACTATTTGTGGCAAGTATAACTGGCCCCGCTTTT	MDIYEAASQGRIDAIKFAVEQGCDVDGPNEDG<br>KTPLWFAVQSGQPEACRFLMSLGAGRGPQNPS<br>LLEVAVGGGYADIVALLWPHCNAEREHRSLKT<br>AISLGFHEIADFLIETGAFEYQDSEVSGTESLIED<br>GSPERESTVFQQWERFLFVRRGQKLPLHRVFFD<br>YALLLATKAGRNAGLRLVEFLLGESMPDVNCK<br>IMINGQFETPLTAAAEKGNLEILATLIDHPNIDL<br>TICGKYNWPAFLHLLASPLSISTERGRVIARRLA<br>YKAVYNRLFIDSREIRLQGAFQNVLRFGDDGLV<br>KQVIDLVRGAAGTLILPLLIRANEVDGLTWVLN<br>CDGVSSKKPPPAFWVLLCQYFKRYQDQDALGL<br>FTSVTEFLVEKKIWNQAILKCLHACNFSFIQQFF<br>YPLSEAPPKEVTEETLSPGFENLLFSNADLNGSD<br>PHPNGLGRLELEIIPHAFFDDPSSAAQKISLRSAL<br>PSASPNPSNPHLYSYQMELIRLEQQNKRRLFFA

	CTCCACCTGCTAGCCAGCCCTCTGTCTATCTCAACAGAAAGAGGCCGT<br>GTCATTGCACGCCGATTGGCTTACAAGGCTGTCTACAATAGGCTGTTTA<br>TTGATAGTCGGGAGATTCGTTTGCAAGGTGCATTCCAGAATGTGCTTCG<br>ATTCGGCGACGACGGCCTTGTGAAGCAAGTAATTGATCTCGTTCGTGG<br>CGCAGCAGGAACTCTTATACTACCCCTTCTGATCAGAGCTAACGAGGT<br>TGACGGTCTGACGTGGGTCTTGAACTGCGATGGGGTCTCTTCAAAGAA<br>ACCACCACCAGCCTTTTGGGTCTTACTCTGTCAATACTTCAAACGTTAT<br>CAAGATCAGGACGCTCTGGGACTTTTCACTAGCGTCACAGAATTCTTG<br>GTAGAAAAGAAAATCTGGAACCAGGCCATTCTGAAATGTCTCCACGC<br>ATGCAACTTCTCTTTCATCCAGCAATTTTTCTATCCATTGTCCGAGGCGC<br>CCCCAAAGGAAGTGACAGAAGAAACACTGGTAGGATTCCCAGCGGCG<br>TCTACTAACCACTGCATCATACAAGAATGGGCTGGCCAAGGGTTTGCC<br>CGTGTCGCTTAGTGGAACGCTATTCACTGTGGATTATGGAAGAGCCCA<br>GGCTTCGAAAATCTTCTTTTTTCCAACGCTGATCTAAACGGGTCAGATC<br>CGCATCCTAATGGCCTGGGGCGACTAGAGCTGGAAATAATCCCACACG<br>CATTCTTCGATGACCCATCTAGTGCGGCGCAAAAGATCTCTCTTAGATC<br>CGCTTTACCTTCTGCGAGCCCCAATCCATCTAATCCGCACCTCTACTCC<br>TATCAAATGGAATTGATTCGCCTGGAGCAGCAGAATAAAAGGCGGTTA<br>TTTTTTGCCGGAGATACGAGGTGCCCCCTTTCTTGGGCCGCGAAAAGCC<br>ACAATGCTCCACTTGTAAATGCTCTGCTACGCAGTCCGCAAGTCAACG<br>TCAATTTTCAGGACCCCTCCGACCGAACTCCGCTTCTGTACGCAATTGC<br>TGTGAATGACAGGCCAATTGTGGAAAGGTTACTGAATCATCGAGATAT<br>TGACCTCAACTTGCGGGATGCAGAAGGCAGAACTGCTATCTTCTACGC<br>CGCACAAGGCGGGGATCTGTCCATTGTACAGTTGCTTATTGGAACACA<br>GAACGTGGACTTTTCTATCCGAAACAAAAACGGGAAAAATGTGAAGG<br>AGTTTGCCAAAAAGGCCAAGTTGAAGCAGGATATTGTGGCTGCACTTT<br>CTAACTGA	GDTRCPLSWAAKSHNAPLVNALLRSPQVNVN<br>FQDPSDRTPLLYAIAVNDRPIVERLLNHRDIDLN<br>LRDAEGRTAIFYAAQGGDLSIVQLLIGTQNVDF<br>SIRNKNGKNVKEFAKKAKLKQDIVAALSN
oryL	ATGCTTTCCTACACTAGCCACTGTCTGCAGGCACTGCTCGGGGTCGCCT<br>CCCTACCCTACCGGCAATACCAGGCGTACTCCAGCCCTCAAGCACCCC<br>TCCAAGTACCCCAAGTACCCCAAGCCGGACCCCCAATCACCACACTGG<br>TCTCGTCATGCGCGGGGTTCAGCTACCCGGAGGTTGCCTGTATTGATCG<br>CTACGGATCCCTCCTGCAAGGCGAGTTCGAGCGCAAAGTACGCAACGT<br>GCTTGGCGACGCCGACACCTACATCTCCACCAACGCGCCGTCAGAACC<br>AACCTTCTCCGATCTGCAGAATGCCGATTTCCTCGTATGGAACCAATCG<br>GCGGCAAAGGCCATCCTGGGTCCCAACCCGCACGTCGATTTCATGTTC<br>AGCATCGAGGACTGCTCCCACGAGGCTCCCGTGTACGTCCCCACTACT<br>AACGAACTGTACTTCTCCCGCTTACAGCAGGGATTCCTCCCTCAACTGG<br>TGATTAACTTGAACAATGACCCTCCTACTTTGGAAGAGAAGCTAGCCC<br>AGCCACCTATTTACGCCGCCACGGGAGCGCGATTCCGTGATGGCCTCC<br>TCTACCTCGCCACGATAGGCGGAAACGAGTCTCTTGCCGGCTACACGT<br>TTCGTCCAGGTCTGTATACGTTGGACCCTATTACGGGAAAAACTCAAG<br>CTCTTCTGAACAACTACTACGGGTATTATTTCAATGCAGTGGACGATCT<br>TGACATCGATCATGAAGGTCAGATCTGGTTCACGGACAATGGTAACAC<br>TGCCTTCCTCCTCCCTACCCTCCCTACTCCACTACCTACCCCCCTATCAA<br>ACAGTCCAGCTAAACAACCAATTCCCAAAACTCGAAAGATTACGGCC<br>GTCCCTGCCAAGTAAACACCTACGCCCCCCAAATCAACGCCGCCACCT<br>ACCGCTTCAACCCCAAAACCGGGCTCGTAACCATGGTCGACGACACCC<br>TCCTCGAACCCAACGGACTCACCTTCTCCCCGGACAACAAAACCGTCT<br>ATCTAACCGACACAGGCGCCGGCTCCGCAATCATCGACCCCAACATCT<br>ACCCAGCACCGCACATCGCCTACAACTCCACGCGCAAAGGCCGCACC<br>ATCTACGCGTACGACGTGGCGCCGTCCCGCAAGGCATTGCTGAATAAA<br>CGCCCCGTGTACTTGTCCATGGAGTATGCGCCGGATGGAATCAAGACT<br>TCCCGGGAAGGGTATTTGGTGTCGGCGACGGGGAAGGGGGTGGTTGTT<br>TTGACGGATGAGGGGGAGCCGTTGGTGAGGGTGCAGACTAATTTCACG<br>GTGATTAATATTGCGTTTGCCGGGGCGGAGAGGGATGAGCTTTGGGCT<br>ATTGGGAAGGGGGGTGTGGCGAGGATTAGGTGGGGGTTGAAGGGGTC<br>TTATGCTTGA	MLSYTSHCLQALLGVASLPYRQYQAYSSPQAPL<br>QVPQVPQAGPPITTLVSSCAGFSYPEVACIDRYG<br>SLLQGEFERKVRNVLGDADTYISTNAPSEPTFSD<br>LQNADFLVWNQSAAKAILGPNPHVDFMFSIED<br>CSHEAPVYVPTTNELYFSRLQQGFLPQLVINLN<br>NDPPTLEEKLAQPPIYAATGARFRDGLLYLATIG<br>GNESLAGYTFRPGLYTLDPITGKTQALLNNYYG<br>YYFNAVDDLDIDHEGQIWFTDNDYGRPCQVNT<br>YAPQINAATYRFNPKTGLVTMVDDTLLEPNGL<br>TFSPDNKTVYLTDTGAGSAIIDPNIYPAPHIAYN<br>STRKGRTIYAYDVAPSRKALLNKRPVYLSMEYA<br>PDGIKTSREGYLVSATGKGVVVLTDEGEPLVRV<br>QTNFTVINIAFAGAERDELWAIGKGGVARIRW<br>GLKGSYA
oryM	ATGACCGTCACAGACTCCACGCCAGAAGGAAACGTCACTGCAGAGCT<br>CTGCAACTGGGTGACAGAGCTCAAGCCCTCAGACATCCCGGCAGATGT<br>CCTTCAACGAGCCAAACATTTGCTGCTCGACGGCATCGCCTGTGGCCT<br>GGTCGGATCTCACGTCCCGTGGTCCGAGCAAGCGGCCAAGGCCATCGA<br>TGACTATGAACCGGAAGGATATTGTAGTGTCATAGGATACAACCGGGT<br>ATCAACTATCCAATATCCACCCAATGAATGATTGACTAACGGCAACTA<br>GCGATACGGCCCCCAAGCAGCGGCCATTCTCAACGGTTCATTCATCCA<br>AGCCGTAGAATTAGATGATTACCACAGTGCCGCACCGCTCCACTCCGC<br>CAGTGTCCTCTTGCCTGCATTATTCGCCGCAGCGGAAGTGCAGTCCAA<br>GGGACACCGGAAGAGCGTGGTCTCGGGACTCGATTTCCTCGTTGCCCT<br>AGTGGTGGGATTCGAAACGGGACCACGAGTCGGATCCGCCATGTACG<br>GTGCAGATCTGCTTTCGCGGGGATGGCACTCAGGTCCGGTCTTCGGCTC<br>TCCGGCAGCGGCAGCTGCGTCCTCGAAGCTCTTGGGCCTCAGCCCAGA<br>TGACACCGAGTCAGCGGTCGGGATCGCCTGCACACAGGCGGGCGGGC<br>TGATGGCAGCACAGTACGAAGGAATGGTGAAGCGTGTACAGCACGCG<br>TTCGCCGCTCGCAACGGACTCTTCGGTGCTCTCTTGGCTCGCGACGGAT<br>ACGTCGGAATCAAGAAGGTGTTCGACCGCAGCTATGGCGGTTTCCTCA<br>CCATGTTCACACAAGGAAACGGCCGGACTCCGCAGTATAAGCCGGAG	MTVTDSTPEGNVTAELCNWVTELKPSDIPADVL<br>QRAKHLLLDGIACGLVGSHVPWSEQAAKAIDD<br>YEPEGYCSVIGYNRRYGPQAAAILNGSFIQAVEL<br>DDYHSAAPLHSASVLLPALFAAAEVQSKGHRK<br>SVVSGLDFLVALVVGFETGPRVGSAMYGADLLS<br>RGWHSGPVFGSPAAAAASSKLLGLSPDDTESAV<br>GIACTQAGGLMAAQYEGMVKRVQHAFAARN<br>GLFGALLARDGYVGIKKVFDRSYGGFLTMFTQG<br>NGRTPQYKPEEVTTALGKEWQTTNIRVKLHAC<br>VGGCHGQIEALEKLQRNYPDRFAVDQLHNIRR<br>ITVSLSEPVFAHDGWAPEERPLTATGGQMNAA<br>YIGAAQLVYGQVLLDQFEPHALDSDAVWSLID<br>KTTCVHSSEFDKPGHLCGARIVVEFNDGETVED<br>VVAMPKGFDPPITDDEIREKWRKLASSVIDSERL<br>QRIENSVLSLETSADVSELLALISGEL

	GAGGTAACGACCGCACTGGGGAAGGAGTGGCAGACGACGAATATCCG<br>CGTGAAATTGCATGCCTGTGTCGGCGGCTGTCACGGACAGATTGAAGC<br>CCTGGAGAAGCTCCAGCGGAACTACCCTGACCGATTTGCTGTCGACCA<br>GCTGCACAATATCCGTCGCATTACGGTTTCCCTGTCTGAGCCGGTGTTT<br>GCGCACGATGGCTGGGCCCCGGAGGAGCGGCCGTTGACTGCGACTGG<br>CGGGCAGATGAACGCGGCGTACATTGGTGCCGCGCAGCTGGTGTATGG<br>ACAGGTGCTGCTCGATCAGTTTGAGCCTCATGCTTTGGACAGTGATGCC<br>GTCTGGAGTCTCATTGACAAAACCACCTGTGTTCATAGCTCGGAGTTCG<br>ATAAGCCAGGTCATCTCTGCGGGGCACGCATCGTGGTTGAGTTTAACG<br>ACGGGGAGACTGTGGAAGATGTGGTTGCCATGCCGAAAGGTTTTGATC<br>CACCCATCACGGACGATGAAATCCGTGAGAAGTGGCGCAAGCTGGCG<br>AGTTCCGTGATCGACTCGGAAAGACTGCAGAGAATCGAGAACTCGGTT<br>CTGTCTTTGGAGACTTCAGCTGATGTGTCGGAATTGTTAGCTCTGATCA<br>GCGGTGAGCTGTAG	
oryN	ATGGCGGTAGCTGAGTTACCCAATATTGTGTCTACGGACTCTTCACCCT<br>CCCCTCATCCGGGTAGTCGACTATCATCGGAACCTACCGACATCGAAA<br>GCCAGAAAGCTCCTTCAAACGCCGAGCCCAAGACGGATCCAAACCTA<br>GTCACCTGGGATGGACCGGATGATCCAGCCAACCCCCAAAACTGGTCC<br>TTCGCATACCGGGCGTTTGTCACAGCCATCTGGGTCTATGGAAACCTCT<br>GCACATGTATCGCCAGTAGTATCTTCAGCAGTGGAAGTGGCCAAATCG<br>CCCAACGGTTTCATGTCGGCAGTACGGTGGTGACACTTGGAATCAGCT<br>TGTTCTTGCTGGTAGGTCGTATCTATCATTCTTTTTTCTTGTCTATGTGAA<br>AGACTGACTAGATAGGGTTACACTGTTGGTCCGCCCGTATGGGGTCCTT<br>TGTCTGAGCGATTCGGGCGTAAGTGGCCCATGGTCATCGGTATGGCTCT<br>TTTTACCATCTTCTGTATACCCGTGGCCGTGGCGAAGAATTTGCAAACG<br>GTCCTCATCGGTCGGTTCCTGACCGGAGTCTTTGGCGCGGCTCCCTTGT<br>CTCTTGTCGGAGGTTCATTGGTGGATATGTGGAATCCCGCGCAACGTGG<br>TGTGGCGATGGCATGTTGTATCGGCACAATCTTTGGCAGTCCGGTGTTG<br>GCCCCTTTGATGGGTAACTTTATCGTGGAAAGCTATCTGGGGTGGCGGT<br>TCACACAATGGCTCAGTTGCATCATGGGCGGGTCATGTACAGTTCTGGT<br>GGTCTTTGGGCTGCCCGAGACATTTGCCGGGTCCATCCTGCGCAAGAA<br>GGCTGCAGCCATCCGCAAGGCGGGCAACCCGGACGTCCACACGGTGT<br>ATGATGGGAAGCAAAAGGGAATCAAGGATATATTCGTCATCTTCTTGC<br>TGAGACCATTTGGTTAGTACACTGTCCCTCGTGTGACTCAAGACATGCT<br>AACAGTGCACAGCACTTTTGGTTACCGAGCCGATTCTTCTGCTGGTCAC<br>TATCTACCAGGTAAGGAAAACCGCTATAGAACATTTACATACGTATGC<br>CCTGACAAATGTACTAGGCTTTCATCTATGGCATCTTATACCTCGTGTT<br>CGTTTCGTACCCTATCGCCTTCCGCGAAGTACGTGGCTGGTCGCTGGGA<br>ATCAGCGCCCTCCCATATATCGGCATGATGGTCGGAATTCTGATCGGTT<br>GTGCCATTGTGGTCATACAAACGCGACGCAACTACGACGGGAACAAA<br>GCCGTGGTCCCCGAGCAACGCCTGCCCCTCATGATCGCGGGAGGATGC<br>CTGCTCCCCGTCGGACTCTTCATCTTCGCCTGGACATCCAACCCCAACA<br>TCCACTGGGCTGGCATGGTCATCGGAAGTGCCCCCGTCGGCACAGGCA<br>TGTACATGGTCTTCGTGCAGTGTCTGAACTACCTAGTTGACGTGTACCC<br>GACCATCGCCAACAGTGCGATCGGCGCCAATACCTTCGTGCGTAGCTT<br>CTTCGGCGCCGGCTTCCCACTCTTCGGACCTTTCATGTATCACAACCTT<br>GGCGTGGCCTGGGCGTCGAGTACCCTCGGGTTCATCAGTATTGCGATG<br>ATCCCGATCCCCGTACTCTTCTATCGGTATGGTGCTCGGATTCGGTCGT<br>GGTCCAAGAATAGCAAGCACACGTGA	MAVAELPNIVSTDSSPSPHPGSRLSSEPTDIESQK<br>APSNAEPKTDPNLVTWDGPDDPANPQNWSFA<br>YRAFVTAIWVYGNLCTCIASSIFSSGSGQIAQRF<br>HVGSTVVTLGISLFLLGYTVGPPVWGPLSERFGR<br>KWPMVIGMALFTIFCIPVAVAKNLQTVLIGRFL<br>TGVFGAAPLSLVGGSLVDMWNPAQRGVAMAC<br>CIGTIFGSPVLAPLMGNFIVESYLGWRFTQWLSC<br>IMGGSCTVLVVFGLPETFAGSILRKKAAAIRKAG<br>NPDVHTVYDGKQKGIKDIFVIFLLRPFALLVTEP<br>ILLLVTIYQAFIYGILYLVFVSYPIAFREVRGWSLG<br>ISALPYIGMMVGILIGCAIVVIQTRRNYDGNKAV<br>VPEQRLPLMIAGGCLLPVGLFIFAWTSNPNIHW<br>AGMVIGSAPVGTGMYMVFVQCLNYLVDVYPTI<br>ANSAIGANTFVRSFFGAGFPLFGPFMYHNLGV<br>AWASSTLGFISIAMIPIPVLFYRYGARIRSWSKNS<br>KHT
oryO	ATGACGGCCCGGACCCCTAACAGGGCGACTGGTGCCCCAGAAAATGC<br>AAATCCCACTGTTCGAGACCAAACGCAGCAGGATCAGGGGGAACGGA<br>GTGAATCGCCAGCCATACCCAGCCTTACCAACAAGAAACGCCCGCGG<br>TCCCAACCAGACAACACCTCCAGTCCTGCCTGCAATCAGTGTCGCACA<br>CGGAAGATTCGCTGCGACCGACAGCAACCAAAGTGTTCCAACTGTCGC<br>CGGGCGGATGTCGAATGTGACTTTGCCACTACCCCGAAGCGCGTCGAC<br>CGCACCAAACAACTCCTAAATGATTTCAGCGGTGTCGTGGCCCGCCTC<br>GATCGGGTGGATAATTCGCTGGCGAAGCTATCGGAGCAGCTGCAGCA<br>ACAGCAGCCATGTCGGTGCTCCCACTCCCCGGTCCCATCGCAGGTGGA<br>TAATCCCTGGGGGGCGTCCGAACCCGCAGCCATATACACCACGTATAC<br>TCCGAAGAGCTCCACCTCGTGCCGATCCCCCCACCTGATGGAGGTGGA<br>CGGTTCCGAAGACTGCGATCCGGAGATTCCCAACGGCGATCTCGTCGA<br>TTTTGACCAGGGCGGCCAACGCCTGCTGGACTACCCGGCCGCATTGTC<br>ACTGTTTAAGAACCTGCAGCGACAGATTACCCGGTGGCTGACCAAGGA<br>TGTGCCGCAAGGATCGGAATTGTGGCAGGTGATCGCCCAGCAACCCGG<br>GTTCAAAGCAAGTTTGGAATATCAGCTGGAGCAGTTTCCCTTTGGCGG<br>CCTCTGTCACGAACCCGTCATCGTGAGTGATCACCGACCCATCAGTAC<br>ACCGCCTCGCTACCTATTAGAGCTATCCTTGGATGGCTTCCTACGCCAT<br>ATCAATATCCATACGCCTATCTTCGACGACTCCTCTCTGGGGAAAGCC<br>ATCGATACCCACTACCAGTCATTGTCCGCTGGAACCGGCGATGCGTGG<br>GCCCTTACTTTTACCAACATCATCATTCTGACGGTAGCCCTGGATGCTC<br>GTGTCGCTCGCGCAACTGCCTCCCATCTAGTCAGCATGAACGATGATA<br>TGCTCCCCTCCTTCCTGAAAAATTGCGATCGAGCATTGGCGGATCTGAA<br>CCGGTTTACTGCGCCATGTCTAATCAATGTTCAGGTGCTCTTGACTCTG<br>GTAAGGATGGTCATCAACTTTCCTGAAGCTTATATACGAGTATAAAAA<br>ATTTTGAAAGCAAGCTAATCCGTTGTGTACAGGCTCTCGTCGCTAGGG	MTARTPNRATGAPENANPTVRDQTQQDQGER<br>SESPAIPSLTNKKRPRSQPDNTSSPACNQCRTRK<br>IRCDRQQPKCSNCRRADVECDFATTPKRVDRT<br>KQLLNDFSGVVARLDRVDNSLAKLSEQLQQQQ<br>PCRCSHSPVPSQVDNPWGASEPAAIYTTYTPKSS<br>TSCRSPHLMEVDGSEDCDPEIPNGDLVDFDQG<br>GQRLLDYPAALSLFKNLQRQITRWLTKDVPQGS<br>ELWQVIAQQPGFKASLEYQLEQFPFGGLCHEPV<br>IVSDHRPISTPPRYLLELSLDGFLRHINIHTPIFDD<br>SSLGKAIDTHYQSLSAGTGDAWALTFTNIIILTV<br>ALDARVARATASHLVSMNDDMLPSFLKNCDR<br>ALADLNRFTAPCLINVQVLLTLALVAREFYGSV<br>VFEKVCQAVCQVARSTGLHRAHGARSMRWEK<br>MPERERLFWVVVYTMDKQRAFLTGQPCDLYLFD<br>SDIQLRSCGERAPFPLRLNAAYVHMMTIWEQIFI<br>NLYSSRAVLAGAADRSRQVQQLWGSLNEWNI<br>KYHALLSSPILEKMADLAPMQLELKYCFMVSQ<br>VLVHRCDRNARSQQRYRDPARSALKLIAQVAG<br>DHRSITLARCAVLARMFRNYPLVAVHDLFSFCL<br>TDGEPDSTEDGQLIHETRRHLELLHYADFPQAY<br>FARLEVGLKWCTDMLDTIKDCLSRSAVAGDW<br>GPMDGSSTGLSDRTPPSESELSSIPPDAWASLNL<br>PMSRDEMLCGLSPSVPSEGLLDPQFSAFGLTTSS<br>TGDSTVPAFAQGGDNPAVTMHPSMWVPAEVP<br>CNSSEPLFDPEFTRSIMS

	AATTCTATGGTAGCGTAGTGTTTGAAAAGGTTTGTCAGGCAGTCTGCCA<br>AGTGGCCCGCTCTACGGGGCTCCATAGGGCTCATGGAGCACGAAGTAT<br>GAGATGGGAAAAAATGCCTGAGCGTGAACGGCTGTTCTGGGTGGTATA<br>CACCATGGATAAGCAGCGAGCCTTCCTAACAGGCCAGCCCTGTGATCT<br>ATACTTATTCGACTCCGATATCCAATTGAGGAGCTGCGGGGAGCGTGC<br>ACCATTCCCGTTGCGGCTCAACGCCGCCTACGTGCATATGATGACGAT<br>CTGGGAGCAGATTTTCATCAATCTCTATTCTTCCCGAGCTGTCCTCGCC<br>GGCGCGGCCGATCGGAGTCGCCAGGTGCAGCAGCTATGGGGTTCATTG<br>AATGAATGGAATATCAAATACCATGCGCTCCTAAGTTCGCCTATTCTCG<br>AGAAGATGGCTGACCTAGCGCCCATGCAACTCGAGTTGAAATACTGTT<br>TCATGGTGAGCCAGGTTCTCGTACATCGCTGTGACCGTAATGCACGCTC<br>CCAGCAGCGGTATCGAGATCCCGCCCGCAGTGCGCTCAAACTCATCGC<br>TCAGGTCGCCGGGGATCACCGTAGCATCACGCTCGCGCGATGTGCCGT<br>CTTAGCCAGGTTTGTTGTTCGATCCTGTCCAGGCTTCCGTGAAGTCGCT<br>GACCACCCCTAGGATGTTTCGAAACTATCCCCTCGTTGCTGTCCATGAT<br>CTTTTCTCTTTCTGTTTGACAGATGGCGAGCCTGACAGCACGGAGGACG<br>GACAACTCATCCATGAGACTCGCCGCCATCTCGAGCTCCTGCACTATG<br>CGGACTTCCCTCAAGCGTACTTTGCCCGACTCGAAGTCGGGCTGAAAT<br>GGTGTACCGACATGCTGGATACCATAAAGGATTGCCTGTCCCGCTCTG<br>CGGTAGCCGGTGACTGGGGCCCGATGGATGGCTCTTCGACCGGCCTCT<br>CCGACCGCACTCCCCCCTCAGAATCTGAGCTCTCGTCCATACCCCCGG<br>ACGCGTGGGCCTCGCTGAACCTTCCGATGAGCCGCGACGAGATGCTTT<br>GTGGCCTTAGTCCTTCAGTACCCTCAGAAGGCCTGTTGGACCCTCAATT<br>TTCTGCCTTTGGATTGACCACGTCGTCTACCGGCGATAGTACCGTCCCG<br>GCGTTCGCTCAAGGCGGTGATAATCCCGCGGTGACCATGCATCCATCA<br>ATGTGGGTACCGGCTGAAGTACCCTGTAACTCGTCTGAACCACTTTTCG<br>ACCCCGAATTTACCCGAAGCATAATGTCATGA	
oryP	ATGATTCTACCTGCAGATCCAATATTTTCGCAATTGCTTCTTATTGCGCG<br>AAACAGCCCTGATGAAGTCGTGATCGATGATCGAAATCTTCATGTCCA<br>GGCAGGCTACAGCCATCTCCTCCATGATGCGGTTCAACTCGCACAGCA<br>GTTGCGAGATAGCCTCTCACAGGGCCCTAGTACTGTCGGTAGTGCTTTT<br>ATTGGGATTCTAGCTCCAACCAGCTATGAATCTACAGTAGCTTCCTTGG<br>CAATTCTCGCCGTTGGAGGTGTGTGTGTGTGCCCATCTGTAAGTTCAAA<br>TCCAAACGTTGCTAGGAATATAAGCGTGCAAGATCTGATTAGGTCAAC<br>AAATTATTACAGCGGCCGGTGCATCTTTAGAGGAATTAGCATATACTTT<br>GAAACAATGTTCAGCCACGTGTGTTTTAGTCGGTTCCCAGCACCAGCA<br>GACAAAACTGGCCACACAACTCCAAGAGCAGACAGGAATCCTCAAAC<br>TTGCGATCCCAGTACTGTCCCCTGGCCGTCCCCCCATTGAGTCGTATAC<br>TCTAGATGAGGATTCAATTCCATCAGACGATTTACCTGCCTTTCTGTTCT<br>TCACTTCCGGGACCACAGGGGCCCCTAAAGGTGTTCTGCATGCTCGAA<br>GGTATTTGTACGCCAAATTCTCAGTCCAACAGTCCGAGTTGACGGATG<br>AGCTCTGTCTGATATATGATTCTATCTGCTGGTCAACCTGTTTTATTTCT<br>GTCCTGCTCCACATTCTGCGGGGGGAGCGAGTCGAGCTACATGAGCTT<br>GACGCCCGCTATGATTTGATCTGGGATCGGTTTCGGGACTGTGAAATCA<br>CCAAGATTCACTTCTCTCCGACCTCTTGGTATACAATGATGAAAGTGTT<br>TCAGGAGCGCATCTCTAAGCTTCCCGAGCCTTCTGTCCAGGCATATATC<br>CGGGGGGCCCAGTACATTCGAACCCCAATTACCTTGGGTGGAATTCTT<br>CCAGTTCCCGTCAAACAGTTCTGGCTGAATCTGCGGGGAGGGCGGCCT<br>ATAAAGGTGATATATGGGAGTACTGAAGCGGGCCTCCTTACTGTGGCC<br>GATCCGGAGGCGTCAGCGAGTGAAGAGGTATGTTACTTATCTGAGACG<br>CTCGTCGCCTCGGGATCCTTGGCTGACCTATTCGAATGTGGTGGTCATT<br>AGGCATCTATTGGGTCACCGGCACCGAATGTCACAGTCAAACTGTCGG<br>ATGGAGATTCCGGAGAGTTGCTTGTCAAAGCTCCAACTCTTCTCTTACA<br>GTAATACTACCCCCCCCGACCCCCTTTATTATTCCTGTACCGCACTACT<br>ATTGCTTTATGCCATGGTGAACCGTACATATGTGTTGACATAGCCTGTC<br>CTCTTCTTTAGATACCTCAACTCTCCGGAGCTCACTGCATCGTGCTTCG<br>ATTCCGAAGGGTTCTATAAGACGGGCGATTTAGTCGAACGACAGGGA<br>AAGAACTTCATCTTCCGAGGTAGATACAAGGCGGACTGTAAGTGTCTG<br>ATCCCGTATCCTCATTCATTGCCATGAACTTGTTATGCCTCATACAAGTT<br>GACATACGGCTGCTGAACCATGTACCTCTAGTTTTCAAATTCTGGGATC<br>ACAAGATCCCTAGGCTTCATGTAGAGTCCTGTCTTTCAAGTCTCCCCTA<br>TATTGAGGAAGCTCATATCCTCCCTGTTGCAGATGCACGATGCGATAA<br>CCGTGTAGCAGCGCTTGTACGTCTTCGCCAGGACCACACGTGTGTTAC<br>ATTACAGAGCATCAGGAAAGATCTCTCAACCATGCTTCCAGTTTATCA<br>AATGCCGACATTGCTTAGGATTTTGGGTAAGGGGGATGAGGTGCCTCG<br>CACATTCTCAGAGAAGGTTGCAATGAAAAAGACTGTGGAGAGGTTCTT<br>TCCCCGATGGAACAATGATCACTTTATGGACGATAGCATAGAAGTGCT<br>GGGCATCAAGGAGATTCTACAGTTCGATACTACAGGCCCATTGGAGCT<br>AGTGGAGCTATGGTAG	MILPADPIFSQLLLIARNSPDEVVIDDRNLHVQA<br>GYSHLLHDAVQLAQQLRDSLSQGPSTVGSAFIG<br>ILAPTSYESTVASLAILAVGAAGASLEELAYTLK<br>QCSATCVLVGSQHQQTKLATQLQEQTGILKLAI<br>PVLSPGRPPIESYTLDEDSIPSDDLPAFLFFTSGTT<br>GAPKGVLHARRYLYAKFSVQQSELTDELCLIYD<br>SICWSTCFISVLLHILRGERVELHELDARYDLIW<br>DRFRDCEITKIHFSPTSWYTMMKVFQERISKLPE<br>PSVQAYIRGAQYIRTPITLGGILPVPVKQFWLNL<br>RGGRPIKVIYGSTEAGLLTVADPEASASEEASIGS<br>PAPNVTVKLSDGDSGELLVKAPTLLLQYLNSPE<br>LTASCFDSEGFYKTGDLVERQGKNFIFRGRYKA<br>DFFKFWDHKIPRLHVESCLSSLPYIEEAHILPVA<br>DARCDNRVAALVRLRQDHTCVTLQSIRKDLST<br>MLPVYQMPTLLRILGKGDEVPRTFSEKVAMKKT<br>VERFFPRWNNDHFMDDSIEVLGIKEILQFDTTG<br>PLELVELW
oryQ	ATGCGTCTTTGGCCAGCCATCGCCATCAGTCTACCCCGTGTGACACCCC<br>CAGAAGGCTGTGAGATCGATGGCTCCTGGGTGCCAGGCGGTACTAAGG<br>TCGGCGTTAGCCAATGGTCCGCATACCGTTCCGAGCGCAATTTCGCGC<br>GAGCCGATCAGTTTCTCCCGGAAAGGTGGTTGCCGGAAGGAGAAGAA<br>GAGTCTTTCATCAATGATACTAGGGCGGCATTTCAGCCATTCAGTACCG<br>GCCCGCGAAATTGCCTGGGAATGAAGTAAGCTATACCTATGCCATGCA	MRLWPAIAISLPRVTPPEGCEIDGSWVPGGTKV<br>GVSQWSAYRSERNFARADQFLPERWLPEGEEES<br>FINDTRAAFQPFSTGPRNCLGMNFARAETRIIFA<br>RLLLDFDLELLTGRDEWEAQKVYIIWDRCPLYV<br>RGFGRQTRHKRPANLWLPWDTHGSSASNERK<br>LADLLS

	GTTCGGGTTAACACCGTTTTACTAGTCGGCTAACCAGATTCCTAGTTTT<br>GCCCGGGCGGAGACTCGAATTATTTTTGCTCGCTTGTTGTTGGACTTTG<br>ATCTAGAGCTTTTGACCGGCCGGGATGAGTGGGAGGCGCAGAAGGTTT<br>ATATCATCTGGGATCGGTGCCCCCTCTATGTTCGGGTCAGACAAGCGA<br>AGAGACGATGAAACACAGGAGCTGCAAAGAATTTATTCTGGAGGTTTC<br>CTAGTCATACTGTCATAGACTTCAACTAAATGCTTCTCCATGTCATTCT<br>ATCATATAAAGCTCATTTATATTTTGGAACCAACTCACTAGAGGCGAA<br>ACCCCCCTTGACGTTTTCTCGGAGTTCCCCATATCGTGGAAAACTCATA<br>TCATTATATTTAAATCTATTCCTTAATTAATGTTGACCCTTTGTGACATA<br>TATTGCCGTAGTTTTATCAACAAAGAGGGGATGTTAATCAATATTCTAT<br>TGCCGAAAGTAGACGTTTCCTTGTCTAGCTGGAGAGGGTCTGACTGAG<br>CCTGCTGAGATAAGATATCAGGAAGGATTTCTTGCTGAAGAATGCAAG<br>CGGCATCCCTTCTGACTGGTGCTTAGATCAGCACGGTCTGAGGGTTGTT<br>TTTTATTTATTTCTTTTTCTGAGTCAGGACGACATGAAATGAGACCATG<br>CACGCGTGAGGAAAAGGTACAACCGGAAGTATGCAGGGTGCACAACA<br>TGTTCACGGAGGACAACAACGCTCCGACGCGAGTAATTTGGCATCCGA<br>AAGTAAATTTTTGGCTGAGACATAGCCCAATTTTGAAGATTTGCAAGCT<br>ACCTACATTCTGAGACATGCCACGCCACGGTACTTAGAGGCTCACCGT<br>CAGCAATGGGTACTATAATGACATGGGATATCACTTTTACCGCCAATC<br>ATTGGTGTCGATGCGTTATATTATCGTATTACCGATACACACTCTGACA<br>CGCTCATGGTGTGGTTTGCCACGTGAGGGTCTAGAAGTGAGCCTGATT<br>GACTGACACTTCAACTGAATGCTTTAATGGGAATCGCCCCGACCCGCA<br>AAACCCCCGTTACGAACACTAAACTATTACTCCTGCAGGGATTCGGTC<br>GACAGACTCGCCACAAGAGGCCCGCTAATCTGTGGTTGCCCTGGGATA<br>CCCACGGCAGCTCGGCTTCAAACGAGAGGAAGCTGGCCGACCTTCTGT<br>CGTAA	
oryfas B	ATGGCCTCCACCCCCAGCTCTGGTTCCTATGATCTTGCGATCGTGACAC<br>CCACGGAAGACCATTCGTTGAGGTCTTTTTCTCTTTCCCAGGGGAACGT<br>CCAGCAAACATTCCTTGTATCGACACAGGACGGTGCTTTCCTGGACCA<br>GCAAAAGGCCTCCTTCCTTCAGAGCTATTCGAAAGACCAGTCAATTCTT<br>GGCTTGGTCTTTGAATTTTTGCAATTCCTGCTGGATGAAGCTTGCCCGCC<br>AGCTCCCCTAGGAGCGTTCCTCGGCGCCATCGAGTCACAGTGCGTGAG<br>AGACGCGAATATCCACGACCTGATTGTGTCCGAGCCAGAGGCCAAGA<br>ATATCATACGCACCTACTACCGCGCACATGCGGTTGCCGGTCTTAACC<br>CCCGTCCAGCCCCCAGTGGACTCTTCAGCACCGTTAATATTGAAGCAC<br>ACCGCATTTTGATGGCCTTCGGGGGCCAAGGGTCTACGAATCTTGTCTG<br>TGTCGACGAACTGGCTGATCTATACTCACTGTACCAACCCTTGCTGGAG<br>CCGCTGATCGCGTCAGTAGCGCCAGCCCTGGCAAGCCTTTCTCGAGAA<br>CCGTCCACGTTGCAGCACTACTTGGGCCGTGAGATTGATCTTTACTCCT<br>GGTTGACTATTCCCGAGTCGCGTCCGGACCGGGCGTTTACAGCTACCG<br>CCGCAGTCAGCTTCCCAATCATTGGCTTACTGGATATGGCGCATTACTA<br>TGTGCTTGGTAAATTACTCGACTCCGATTCCCCGAAGCGACTAAGGTC<br>AGCGCTCCAAGGCCTCACGGGCCACTCGCAAGGAATTATCGTGGCTGC<br>AGCCGTCGCTCAAGCCGACACGTGGGCGTCCTTTTTGGCGCAGGCACA<br>ATGGGCCATCCGCCTATTGTTTTGGATGGGTTATGAATGCCACACAGCC<br>GCTCCTGCTTCTCCCCTCTCATCCGCCGCCATCAGGGATAGTATTGAGC<br>ACGGCGAGGGATCCCCCTCATGGCTCCTTAGTGTCCGAGGCCTGCGGT<br>CGCCCGCTCTTGATGCGTTAATCACGGACTGCAATCGCCGTTTACCAGA<br>ATCCGAACACCTGTCCATCGGATTGATCAACACTGAGCGGAACATTGT<br>GGTAGCCGGTTCTCCACGTTCCCTCCGCGGCTTATGTTTGCGGTTGCGA<br>GAAATTGAAGCCGATGATGGTCAAGACCAAAGTCGGGTTCCTTTCCGT<br>CAGCGCAAGCCTGTTGTCCATCATACCTTCCTTCCTGTTAGCGCTCCTTT<br>CCATTCCAGCCACCTCCGTGCCGCTGCAGACCGCGTCAAGGAACGGTT<br>TCCGGATGCCTCATCCCCCCAAGTGGGGGATCTCCTCACTGCTGTTTAT<br>CATACACGGACCGGCCAGGACATGCGCGAGATGTTCAGCCCATCGAA<br>TAACCTGATCCATAGCCTGGTCGAGGCAGTAGCCTGTGAGACCGTCGA<br>TTGGCCAGCAACCTTGCAAGTATCACGTTCCAAGCCGCCTTCCCACATT<br>GTGCTTCTCAGCAGCAGCCGTCTCAGTGATCTCGTATCCGAAATTGTCG<br>ATGGCCGTGGAGTTCGTATCATCGCAGGAACAGTGCTAGCCCCAACGG<br>ACCCCGCCGTTCTCGGTGGGAAAGCGGAGTTACTAACCACGAAGCCCT<br>CACAGGCGCCAACCCCTTGGGCGGAGCTCTTTAAAGCTCGACTCGTGG<br>CCGGCCCAGACGGCAGGCCCATACTTGAGACGCGACTCAGTCAGTTGC<br>TGCAAGCGCCCCCTATCATCACAGCTGGTATGACACCCACCACCGTAC<br>ACTGGGACTTCGTGGCCGCGGTGATGCAGGCTGGCTATCACGTTGAGC<br>TTGCCGGCGGTGGTTACTTTGACGCGGCTGGCATGACAACAGCTATTG<br>AGAAACTAGCGGCCCACGTTCCCCCCGGCCGCGGCATCACCTGCAATC<br>TCATCTATGCCAGCCCTCATTCGATTGCGTTTCAGATCCCCCTAATTCG<br>CAGCATGATCCAACGCGGAATCCCCATAGATGGCTTGACAATTGGAGC<br>GGGAGTTCCCTCGCAAGATGTGGTCAACGAATGGATCCAAACACTGGG<br>GATCAAACACCTTTCCCTCAAACCAGGGTCCATCGCTGCTATATATGA<br>GGTGATTGAAATCGCTAAAAAGCACCCGACCTTCCCCATTATTTTACA<br>GTGGACCGGAGGCCGCGGTGGTGGCCATCACTCCTGTGAGGACTTCCA<br>CGAACCACTACTGCAGACCTATCGGGATATCCGCCGCTGTTCCAATTTG<br>TATCTGGTTGTGGGCAGTGGGTTTGGCCAGGCGGACCAGATGCACCCC<br>TATATCACGGGTGAATGGTCGCTGTCATTCGGTCGTCCCGTAATGCCCT	MASTPSSGSYDLAIVTPTEDHSLRSFSLSQGNVQ<br>QTFLVSTQDGAFLDQQKASFLQSYSKDQSILGL<br>VFEFLQFLLDEACPPAPLGAFLGAIESQCVRDA<br>NIHDLIVSEPEAKNIIRTYYRAHAVAGLNPRPAP<br>SGLFSTVNIEAHRILMAFGGQGSTNLVCVDELA<br>DLYSLYQPLLEPLIASVAPALASLSREPSTLQHYL<br>GREIDLYSWLTIPESRPDRAFTATAAVSFPIIGLL<br>DMAHYYVLGKLLDSDSPKRLRSALQGLTGHSQ<br>GIIVAAAVAQADTWASFLAQAQWAIRLLFWM<br>GYECHTAAPASPLSSAAIRDSIEHGEGSPSWLLS<br>VRGLRSPALDALITDCNRRLPESEHLSIGLINTER<br>NIVVAGSPRSLRGLCLRLREIEADDGQDQSRVPF<br>RQRKPVVHHTFLPVSAPFHSSHLRAAADRVKE<br>RFPDASSPQVGDLLTAVYHTRTGQDMREMFSPS<br>NNLIHSLVEAVACETVDWPATLQVSRSKPPSHI<br>VLLSSSRLSDLVSEIVDGRGVRIIAGTVLAPTDPA<br>VLGGKAELLTTKPSQAPTPWAELFKARLVAGP<br>DGRPILETRLSQLLQAPPIITAGMTPTTVHWDFV<br>AAVMQAGYHVELAGGGYFDAAGMTTAIEKLA<br>AHVPPGRGITCNLIYASPHSIAFQIPLIRSMIQRGI<br>PIDGLTIGAGVPSQDVVNEWIQTLGIKHLSLKPG<br>SIAAIYEVIEIAKKHPTFPIILQWTGGRGGGHHS<br>CEDFHEPLLQTYRDIRRCSNLYLVVGSGFGQAD<br>QMHPYITGEWSLSFGRPVMPCDGILIGSRMMV<br>AREAHTSPQAKELILAAAGVADSEWEQSFKKP<br>TGGVLTVQSEMGQPIHKLATRGVRLWHEMDK<br>TIFSLPRDKRVAALNARKAEIIRRLSADFAKPWF<br>GYNAAGDAVDLEDMTYTEVIARLIRLVYVSHQ<br>HRWIDPSYRQLVLDFTYRTLERVSNADYATDKL<br>DLSQPEQFVEQVQQLCPAATTRRLHPDDVRFFL<br>TICKQRGRKPVNFIPALDEDFEYWFKKDSLWQS<br>EDVDSIIDQDADRVCILQGPVAVQYSRRADQSA<br>REILDEIHHGLANHFEEGPSQSDRPSLAISEMVS<br>ARVTVTESNTHRIIRPTSESLPSVEDWQAFLASQ<br>VTGSVRSAIMAEEVLRGSQRQANPLRRVLEPRT<br>GQSIQIPLDGRDLRLVEDAKNRPLVHIKPSGDQ<br>EVAVDFYYYDFVETPGNLRFTYKFDSKSLSLVEN<br>LDGRDDRVKLFYAHLWLGRADLSYHRLSEVFE<br>GEEITLSSDLHRHLHNALRHTVPDATASATTNT<br>LPLEAAIIAAWKPLMEPLFVAELQGDLLRLVHL<br>SNSIRYTPGAAPLEVNDVVATKSQVRAVTIKET<br>GKTISVEAQIFRSKTLVATVTSEFFIKGSFSDYETT<br>FSHQDEAAIELKVQSAIDEALLRDREWFLLDDP<br>TQSLIDKTLVFRLHTVTRWKDQSTFTSLKTTGSI<br>YTKHWNGTEQKVGTVASEVVECHGNPVIDFLQ<br>RKGTVVQEKVPLKHPGLIDNGSRTIRLPLDNAL<br>YSSVSKDYNPIHTSSVFARFADLPGTITHGMYTA<br>AVSRAVTECLAADGETGRLRSFSASFVGMVLPG<br>DQLTVRIRHEAMCHGRMVLSVAAYREGTDEK

	GTGATGGTATCCTCATTGGCAGTCGGATGATGGTCGCCCGGGAGGCGC<br>ACACATCTCCGCAAGCAAAGGAATTGATCTTGGCAGCTGCCGGAGTGG<br>CCGACTCGGAATGGGAACAGAGCTTCAAGAAGCCTACCGGTGGAGTG<br>TTGACCGTTCAGTCGGAGATGGGTCAGCCGATCCACAAGCTGGCAACT<br>CGTGGAGTTCGGTTGTGGCATGAGATGGATAAGACTATCTTCAGCTTGC<br>CTCGGGACAAACGAGTCGCTGCACTCAATGCACGCAAGGCTGAAATT<br>ATACGTCGTTTGAGTGCTGATTTTGCCAAGCCGTGGTTTGGCTACAATG<br>CTGCCGGTGATGCCGTCGATCTAGAGGACATGACATATACTGAAGTCA<br>TTGCTCGTTTGATCCGGCTAGTGTACGTTAGCCATCAGCACCGCTGGAT<br>TGACCCATCCTACCGTCAGCTCGTTTTAGACTTTACCTACAGAACGCTA<br>GAACGTGTCTCCAACGCCGATTATGCGACGGATAAGCTTGACCTCAGT<br>CAGCCGGAACAATTCGTCGAGCAGGTGCAACAACTCTGCCCCGCTGCA<br>ACTACACGTCGTCTACACCCAGACGATGTCCGCTTCTTTCTCACTATCT<br>GCAAGCAGCGTGGTCGAAAGCCCGTCAACTTCATCCCAGCGTTGGATG<br>AAGACTTCGAATACTGGTTCAAGAAAGATTCCCTGTGGCAGTCAGAGG<br>ATGTGGATTCAATCATCGACCAGGACGCTGACCGAGTATGTATCCTAC<br>AAGGCCCGGTGGCCGTGCAATATTCGCGTCGGGCAGATCAGTCAGCCA<br>GGGAAATCCTTGATGAGATCCACCATGGGCTCGCAAACCATTTTGAGG<br>AGGGTCCGTCTCAGTCGGATAGGCCTTCGTTAGCCATATCGGAAATGG<br>TCTCCGCTAGGGTCACAGTGACAGAAAGCAATACCCATCGTATCATCC<br>GTCCTACATCTGAAAGTTTGCCCTCTGTTGAAGACTGGCAAGCATTCCT<br>GGCTAGTCAAGTCACAGGTAGCGTCCGAAGCGCTATTATGGCTGAGGA<br>AGTCCTACGAGGTTCTCAGAGACAAGCGAATCCTTTGCGGCGAGTTCT<br>GGAACCACGGACAGGGCAATCAATCCAGATTCCTCTGGACGGTCGCG<br>ATCTACGTCTGGTAGAGGATGCGAAGAACCGTCCGTTGGTACACATCA<br>AGCCCAGTGGTGATCAGGAGGTTGCCGTAGACTTCTATTATTATGACTT<br>CGTGGAGACCCCTGGCAACCTTCGATTCACGTACAAGTTTGACTCAAA<br>GAGCCTTTCCCTGGTAGAAAATCTCGACGGGCGCGATGACCGAGTTAA<br>ATTGTTCTATGCCCATTTGTGGCTCGGTCGTGCCGATCTCAGTTACCACC<br>GGCTGAGTGAGGTCTTCGAGGGCGAGGAAATCACCCTTTCTTCTGATCT<br>TCATCGTCACCTTCACAACGCTCTCCGTCATACAGTCCCAGATGCCACT<br>GCATCCGCCACAACTAACACCTTACCCCTCGAGGCCGCGATTATTGCC<br>GCTTGGAAACCCCTGATGGAACCACTATTTGTGGCGGAGCTCCAGGGC<br>GACTTGCTCCGCCTAGTGCATCTCTCCAATAGCATCCGGTACACCCCTG<br>GCGCTGCTCCACTAGAAGTCAACGATGTTGTCGCGACCAAATCACAAG<br>TGCGTGCAGTGACCATCAAGGAGACCGGCAAGACGATCAGTGTTGAA<br>GCACAGATCTTTCGGTCTAAGACGCTCGTGGCCACTGTGACTTCGGAGT<br>TTTTTATCAAGGGATCTTTTTCCGACTACGAGACCACCTTCAGTCACCA<br>AGATGAGGCCGCGATTGAACTAAAGGTACAGTCTGCCATAGACGAAG<br>CCCTTCTACGGGACCGTGAATGGTTTCTCTTGGATGACCCTACCCAGTC<br>ATTGATTGATAAAACCCTCGTCTTCCGTCTACACACCGTGACCAGGTGG<br>AAGGACCAATCTACCTTCACCAGCCTGAAAACCACTGGCTCGATCTAC<br>ACCAAGCATTGGAATGGTACCGAGCAGAAAGTGGGAACCGTTGCATC<br>TGAGGTGGTTGAATGTCATGGCAATCCTGTTATCGACTTCCTGCAGCGC<br>AAGGGCACTGTGGTGCAAGAAAAAGTGCCTTTAAAGCATCCAGGCCT<br>GATAGATAATGGATCGAGGACCATCCGCTTACCGCTTGATAATGCCCT<br>GTACAGCTCCGTATCCAAGGACTACAATCCCATACACACCTCGTCCGT<br>CTTTGCGCGTTTCGCAGACCTTCCCGGGACCATCACCCATGGCATGTAT<br>ACCGCCGCTGTTTCCCGCGCCGTCACGGAATGTCTGGCAGCGGACGGC<br>GAAACGGGTCGGCTACGAAGCTTCTCGGCGTCTTTCGTTGGGATGGTGT<br>TACCAGGCGACCAACTGACGGTCCGTATCCGACATGAGGCTATGTGTC<br>ACGGCCGCATGGTGCTCTCCGTAGCGGCATACCGGGAGGGTACCGATG<br>AGAAGGTGCTTCAGGGGGAGGCCGAAGTTGAGCAGCGCACTTCCGCG<br>TACCTCTTTACCGGTCAGGGAAGCCAGGCACAGAACATGGGAATGCA<br>GTTGTATGACTCAAGCGCAGTGGCCCGATCGGTGTGGGATGAGGTTGA<br>CCGCCGTCTGCTGGATCAATACGGTATGTTGTCTCTAATCATTCACATC<br>CCTGAAGCCCTACCTCTTTCCACGGCTCCAGTGGCAGCCTTCCGAATCT<br>GGCACTAACATATCTAGGCTGGTCAATTTTGAACGTTGTCCGCGCAAA<br>CCCCAAACAGATCACTATCCACTTCCGTGGGGCTCGTGGCAGACGCAT<br>CCGCGACAACTATCTAGCGATGAGGACCGAAACCCGAATGCCAGACG<br>GCTCCACTCGGCTCGAACCTATCCTTCGCGATCTAACGGCCAAATCAG<br>AGTCATATACCTTCTTCGACTCCCGAGGCCTGCTGTATGCCACGCAATT<br>TGCTCAGCCCGCTATTCTGCTAATGGAAAAGGCGGCATTCGAGGATAT<br>GAAGGCAAATGGCCTCATCCAGGAGGGCGCTGCCTTCGCAGGACACT<br>CTCTGGGTGAGTACGGAGTCCTAGCCTCATTGGTTGACTTCTTGCCCTTT<br>GAAATGATGATGAGTGTCGTTTTCTATCGTGGGTTAGTCATGCAGTTCA<br>CCATGGAGCGCGACTCCAACGGACACACGGGTTTCTCTATGGTGGCAG<br>TCAGTCCGAAGAGGGTGGGCAAATGTAAGTTTTGTTCTAGTTTCGATCA<br>ACTTTCCCAGGCTCACGGCGAGACAGATTTCGATGAAGCCATGTTACG<br>CATCGTTGTGGATTTGATTCACCGACAGAGTGGCAAACTGTTGGAAAT<br>CGTCAATTTCAATGTGGAAGCCGAGCAATATGTTTGTGCCGGACACGT<br>AAGTTCTCAGCCCCGTTATATCTACCACATCTCGCTCATGCATTCCAGG<br>TCCGCAATATTTACATTTTGAGCGGCATCCTCGACCTTCTGTCTCGATCC<br>GCCACTGGTCCCCAGCTCGTAGCCTCACTCCGCAGCGCTTCTGATCCGG<br>CCATCACTGACGTAGCCAAAGAAATTGCTGTCTATCTCGAAAAGGCAC	VLQGEAEVEQRTSAYLFTGQGSQAQNMGMQL<br>YDSSAVARSVWDEVDRRLLDQYGWSILNVVRA<br>NPKQITIHFRGARGRRIRDNYLAMRTETRMPDG<br>STRLEPILRDLTAKSESYTFFDSRGLLYATQFAQP<br>AILLMEKAAFEDMKANGLIQEGAAFAGHSLGE<br>YGVLASLVDFLPFEMMMSVVFYRGLVMQFTME<br>RDSNGHTGFSMVAVSPKRVGKYFDEAMLRIVV<br>DLIHRQSGKLLEIVNFNVEAEQYVCAGHVRNIY<br>ILSGILDLLSRSATGPQLVASLRSASDPAITDVAK<br>EIAVYLEKAPQLNNPTELKRGRATIPLQGIDVPF<br>HSSHLRSGVSVYRRFLEERIQAENVQVDRLVGK<br>FIPNVMGKPFAIDRSYLEEAAAVTGSSVLRELAL<br>AA

	CCCAGCTGAACAATCCCACTGAATTGAAACGAGGCCGAGCAACCATC<br>CCGTTGCAAGGCATCGACGTTCCCTTCCATTCGTCCCACCTGCGCTCCG<br>GGGTGTCCGTCTACCGACGATTCTTAGAGGAGCGGATCCAAGCAGAGA<br>ATGTACAGGTGGACCGTCTAGTGGGCAAATTCATCCCCAATGTCATGG<br>GGAAGCCGTTCGCGATTGATCGGTCATATCTAGAAGAGGCCGCAGCAG<br>TCACAGGGAGTTCTGTGTTGAGGGAGCTGGCGCTAGCCGCTTGA	
oryR	ATGACTATTCCAGCGGCGGACGACAATAATTGCCCTTCCTATGACAAG<br>GTAATCGATTTGATCGTCGACTACGCCTATGACTATGAAATCGACTCCC<br>CAGCGGCCTGGACAAGAGCCAAAGCAGCTCTTATTGATGCCCTAGGTG<br>CTGCTATCGAAAGTATCCATACTAGCCCAGAATGTGCCGCCATGATTG<br>GCCCCGTCTGGCCCCAGACGGCCACGGTGCCAGGGGGCTTCCGCTTGC<br>CGGGTACTCAATTTCAAGTGGATGCCCTCAAGGGTGCCTTTGACCTTGG<br>GGGAATGATCCGGTACCTTGACCACAACGATGCCTTTCCCGGAGCGGA<br>ATGGGGCCATCCATCCGGTAAGTTAATACCCACTTCTACCGTCTGTCTG<br>TCCTGACCAATGACCAGATAATCTTGGCGCAATCCTCAGCACTGCCGA<br>CATCCTCAGTCGGGAGGCCCTCGCTCGTGGCAGCCCCGAGGAAGTCAT<br>CTCCATGAAGCAGGTCCTCACTGCTCTGATCAAGGCGTACGAGATCCA<br>AGGGGTGTTCCAGATCCGCAACGCGTTCAACAAGGTTGGCCTGGATCA<br>TGTCATTTTGGTCAAGGTTGCCTCATCTGCCATGGTCTCCTGGCTCATGG<br>GTCTATCCCGCGATCAAGCCCGGGCTGTGGTCTCGCATGCCTGGGCTG<br>ATGGTCATCCGCTGCGGGTCTATCGACAGGCGCCAAATGCAGGACCTC<br>GGAAAGGATGGGCCGCTGGGGATGCCTGCATGCGTGCTGTGCATTTGG<br>CCAACCTGGTTCGATGCGGCCAGCCCGGTATCCGCTCTGCTATCACCA<br>CCCCGCGTTGGGGGTTTTATGACGTCTTGTACCGGGGTCAGACCTTCGA<br>GCTACCCCGTCCGTTTACTAGCTGGGTCATGGAGACAGTCCTCTTCAAG<br>GTCTCGACGGCTGAAGGGCACGGACTGACCGCTGTGGAAGCAGCATT<br>GACTATTGCAGAAAAGCTGGCTCAACGTGGGCTTCGGCCAGAAGAGG<br>ACATTGTCAACATACGAGCTCGAACACAGGAGGCTGGAATGATTATCA<br>TCAATAAGAAGGGGCCGCTGCATAATGCCGCAGATCGCGACCATTGCC<br>TGCGTTATATGGTCGCCGTGGTCCTGCTCAAGGGCAGTCAAATCACAA<br>CTGCTGACTACCAGGACAGCAGTCCCTGGGCTCGTGACCCTCGTGTAG<br>AAACACTACGCTCCATCACCACAATGGAAGAAGATCCGTCATTCACCC<br>GGGATTACCATGATCCTCAGTGCCGCAGCGTGGCCAACGCATTGGAGG<br>TGACGCTACGGGATGGCACCAAGTTGGAAGAATTGGTCCCGTTCCCCT<br>TGGGTCATGTGCGTCGACCTGAGACTTTGCAGCTGGTCCGCGAGAAGG<br>CCCAGCAGAACTTGGGTTTGAAGCTGTCGTCGGAGAGGGTGGGGCAG<br>ATATTGGACACGGTCGATCAACCGAAGTTCGAGAAGATGGCCGCTAGT<br>GATTTTGTAGACCTATTCATCCCACAACCTGCGTCCAGCGCTGCATGA	MTIPAADDNNCPSYDKVIDLIVDYAYDYEIDSP<br>AAWTRAKAALIDALGAAIESIHTSPECAAMIGP<br>VWPQTATVPGGFRLPGTQFQVDALKGAFDLG<br>GMIRYLDHNDAFPGAEWGHPSDNLGAILSTAD<br>ILSREALARGSPEEVISMKQVLTALIKAYEIQGVF<br>QIRNAFNKVGLDHVILVKVASSAMVSWLMGLS<br>RDQARAVVSHAWADGHPLRVYRQAPNAGPR<br>KGWAAGDACMRAVHLANLVRCGQPGIRSAIT<br>TPRWGFYDVLYRGQTFELPRPFTSWVMETVLFK<br>VSTAEGHGLTAVEAALTIAEKLAQRGLRPEEDI<br>VNIRARTQEAGMIIINKKGPLHNAADRDHCLR<br>YMVAVVLLKGSQITTADYQDSSPWARDPRVET<br>LRSITTMEEDPSFTRDYHDPQCRSVANALEVTL<br>RDGTKLEELVPFPLGHVRRPETLQLVREKAQQN<br>LGLKLSSERVGQILDTVDQPKFEKMAASDFVDL<br>FIPQPASSAA
